# Unraveling the Photoprotective Response of Lichenized and Free-Living Green Algae (Trebouxiophyceae, Chlorophyta) to Photochilling Stress

**DOI:** 10.3389/fpls.2017.01144

**Published:** 2017-07-04

**Authors:** Fátima Míguez, Ulf Schiefelbein, Ulf Karsten, José I. García-Plazaola, Lydia Gustavs

**Affiliations:** ^1^Department of Plant Biology and Ecology, University of the Basque Country (UPV/EHU)Bilbao, Spain; ^2^Independant ResearcherRostock, Germany; ^3^Applied Ecology and Phycology, Institute of Biological Sciences, University of RostockRostock, Germany

**Keywords:** carotenoid, high light, lichen, low molecular weight carbohydrates, low temperature, pigments, violaxanthin cycle

## Abstract

Lichens and free-living terrestrial algae are widespread across many habitats and develop successfully in ecosystems where a cold winter limits survival. With the goal of comparing photoprotective responses in free-living and lichenized algae, the physiological responses to chilling and photochilling conditions were studied in three lichens and their isolated algal photobionts together as well as in a fourth free-living algal species. We specifically addressed the following questions: (i) Are there general patterns of acclimation in green algae under chilling and photochilling stresses? (ii) Do free-living algae exhibit a similar pattern of responses as their lichenized counterparts? (iii) Are these responses influenced by the selection pressure of environmental conditions or by the phylogenetic position of each species? To answer these questions, photosynthetic fluorescence measurements as well as pigment and low molecular weight carbohydrate pool analyses were performed under controlled laboratory conditions. In general, photochemical efficiency in all free-living algae decreased with increasing duration of the stress, while the majority of lichens maintained an unchanged photochemical activity. Nevertheless, these patterns cannot be generalized because the alga *Trebouxia arboricola* and the lichen *Ramalina pollinaria* (associated with *Trebouxia* photobionts) both showed a similar decrease in photochemical efficiency. In contrast, in the couple *Elliptochloris bilobata*-*Baeomyces rufus*, only the algal partner exhibited a broad physiological performance under stress. This study also highlights the importance of the xanthophyll cycle in response to the studied lichens and algae to photochilling stress, while the accumulation of sugars was not related to cold acclimation, except in the alga *E. bilobata*. The differences in response patterns detected among species can be mainly explained by their geographic origin, although the phylogenetic position should also be considered, especially in some species.

## Introduction

One of the most challenging stressors for photosynthetic organisms living in temperate regions is photochilling. Photochilling occurs when there is a combination of low temperature (LT) and high light (HL) stresses (Huner et al., 2003; Ivanov et al., 2003). Under these conditions, there is an imbalance between the light absorbed and utilized, provoking the over-excitation of the photosynthetic apparatus and increasing the risk of photooxidative damage. To tolerate this combined stress, photosynthetic organisms need protective physiological and morphological mechanisms. Otherwise, they are unable to maintain the balance between efficient light harvesting, photochemistry and photoprotection and are unable to avoid the damage provoked by the excess light (Waters, 2003).

Cold acclimation (CA) is a complex, multistep process involving a series of concerted physiological and biochemical changes (Guy, 1990; Thomashow, 1999). In many species, from conifers (Adams et al., 2013) to algae (Bohnert and Sheveleva, 1998), the CA process is associated with the accumulation of soluble sugars, particularly sucrose. Current models propose that sugars contribute to the acquisition of freezing tolerance, acting as compatible osmolytes, cryoprotectants, scavengers of reactive oxygen species (ROS) and signaling molecules. CA also induces an increase in the activity of antioxidant enzymes as well as an augmentation of the pools of contents of non-enzymatic antioxidants, such as tocopherols and carotenoids. The xanthophyll cycle (V-cycle) is one of the most important photoprotective mechanisms in vascular plants as well as in green and brown algae (Yamamoto et al., 1962; Stransky and Hager, 1970). It consists of a forward reaction comprising two de-epoxidation steps, in which the di-epoxy xanthophyll violaxanthin (V) is converted to the epoxy-free zeaxanthin (Z). The intermediate product of this reaction sequence is antheraxanthin (A), which contains one epoxy group. Z takes part in the dissipation of excess excitation energy as heat, preventing the inactivation and the damage of the photosynthetic apparatus. This process is known as non-photochemical quenching (NPQ) (Demmig-Adams et al., 1990).

As noted recently (Valledor et al., 2013; Míguez et al., 2015), the process of winter photoinhibition and CA has been widely described in higher plants, but little is known about photosynthetic responses to LT of free-living and lichenized green algae. In polar and alpine ecosystems, terrestrial green algae are the most abundant primary producers or even the unique ones (Gray et al., 2007; Büdel and Colesie, 2014; Quaas et al., 2015). These ecosystems provide habitat for a heterogeneous assemblage of microscopic organisms belonging primarily to the Chlorophyta or the Streptophyta (Rindi, 2011). Green algae can live in an aposymbiotic state (not lichenized) or in symbiosis. Lichens are symbiotic associations consisting of a fungus (the mycobiont), a photosynthetic partner (the photobiont) and a diverse bacterial community (Grube et al., 2012). Within the Chlorophyta, the class Trebouxiophyceae is composed of mostly terrestrial algae, including certain genera that predominantly form a lichen symbiosis (Macedo et al., 2009). In general, these algal species have to tolerate extreme light, nutrition and temperature conditions in comparison with their aquatic relatives (Remias et al., 2010), as they typically live in rough terrestrial habitats such as soil crusts (Karsten and Holzinger, 2012) or biofilms (Quaas et al., 2015).

The protective mechanisms of algae against challenging habitats are highly diverse. In the present work, we focused on members of the Trebouxiophyceae, such as *Trebouxia* and *Asterochloris*, which are the most common eukaryotic photobionts (Tschermak-Woess, 1988) that rarely occur in the aposymbiotic (non-lichenized) state (Ahmadjian, 2002; Škaloud et al., 2015). In contrast, other terrestrial algae, such as members of the genus *Apatococcus*, are predominantly free-living (Ettl and Gärtner, 2014), and, although they live in close associations with fungi, a true lichenization is extremely rare (Voytsekhovich, 2013). Representatives of the *Elliptochloris* clade (e.g., genus *Elliptochloris*) are considered facultative photobionts, as they also occur frequently in a free-living state in both terrestrial and aquatic habitats. In terms of biogeographic distribution, there are also differences between the algal genera. *Asterochloris* and *Trebouxia* are distributed worldwide (Ettl and Gärtner, 2014). In contrast, *Elliptochloris* is far less frequent (Gustavs et al., 2017) and might be more sensitive to variable abiotic conditions. Consequently, the plasticity of photobiont physiology in response to certain abiotic stresses such as photochilling has to be evaluated to better understand the benefit that the algal partner might gain from the lichen symbiosis (Sadowsky and Ott, 2015).

Although a high physiological variability in the Trebouxiophyceae has been described (Rindi, 2007, 2011; Holzinger and Karsten, 2013; Darienko et al., 2015), it is unknown if the different lifeforms evolved in response to the selection pressure of environmental conditions at their place of origin or are influenced by the phylogenetic position of each species. Despite the ecological relevance of alpine, Arctic and boreal ecosystems, the respective algal responses to low temperatures have not been characterized in detail. Therefore, the main objective of the present study was to identify general patterns of acclimation in symbiotic and free-living Trebouxiophyceae under chilling and photochilling conditions. To achieve this goal, we analyzed the response of three algae genera (that facultatively live as photobionts in lichens) in free-living and lichenized states and compared them with an aposymbiotic genus. Another goal was also to evaluate if lichenization is beneficial for the investigated photobionts or if they perform equally well in a free-living state.

## Materials and methods

### Organisms and culture conditions

Four terrestrial unicellular green algae and three lichens were investigated in this study (for details, see Table 1). *Apatococcus lobatus* (*AL*), *Asterochloris erici* (*AE*), and *Trebouxia arboricola* (*TA*) were grown on solid (1.5% DIFCO agar) TOM medium (Trebouxia organic medium) according to Ahmadjian (1967), modified after (Friedl, 1989) by the addition of 1.5% glucose, 2% proteose-peptone to 3N-BBM+V. *Elliptochloris bilobata* (*EB*) was grown in-modified Bolds Basal Medium (3N-BBM+V; medium 26a in Schlösser, 1997).

**Table 1:** Taxonomic assignments, habitat and origin of studied species.

	**Species**	**Taxonomic assignment**	**Habitat characteristics**	**Origins**	**Light and temperature in their native habitats**
ALGAE	*Apatococcus lobatus* SAG 2145 (*AL*)	Trebouxiophyceae, *Apatococcus*-clade	Bark of *Platanus* sp. Terrestrial free-living green algae.	Free-living. Switzerland, Basel, Rheinweg,	
	*Asterochloris erici* SAG 32.85 (*AE*)	Trebouxiophyceae *Trebouxia*-clade	Soil	Photobiont of lichen *Cladoniacristatella*. United States, MA, Whitensville	
	*Elliptochloris bilobata* SAG 245.80 (*EB*)	Trebouxiophyceae-*Elliptochloris* clade.	Unknown	Phycobiont of *Catolechia wahlenbergii*. Austria, Kärnten, Kreuzeckgruppe, 2200 m	
	*Trebouxia arborícola* SAG 219.1 (*TA*)	Trebouxiophyceae *Trebouxia*-clade	Unknown	Typical photobiont of *Ramalina* sp. (associated lichen not documented)	
LICHENS	*Cladonia squamosa* (Scop.) Hoffm. (*CS*)	Class Lecanoramycetes	Strongly degraded peat bog, on rotten pine stump	54°03'25”N, 12°24'29”E Sanitz, Mecklenburg-Western Pomerania, Germany	Average annual temperatures: 7.5 and 8.0°C. half-shaded
	*Baeomyces rufus* (Hudson) Rebent (*BR*)	Class Lecanoramycetes	Steep slope at the forest edge, on soil	14°11'30"E/53°54'41”N Korswandt, Isle of Usedom, Mecklenburg-Western Pomerania, Germany	Average annual temperature: 7.5 to 8.0°C. half-shaded
	*Ramalina pollinaria* (Westr.) Ach. (*RP*)	Class Lecanoramycetes	Northern side of rural medieval church, on granite	54°10'27”N, 12°14'31”E Rövershagen, Mecklenburg-Western Pomerania, Germany	Average annual temperature: 7.5 to 8.0°C. no direct solar radiation

*Algal strains (prefix SAG) were obtained from the culture collection of algae, University of Götttingen (Germany). For more information about the ecology of the lichen species see Müller-Westermeier et al. (1999)*.

The lichens studied were *Ramalina pollinaria* (*RP*) associated with *Trebouxia, Cladonia squamosa* (*CS*) associated with *Asterochloris* and *Baeomyces rufus* (*BR*) associated with *Elliptochloris*. They were sampled in October 2014 in different localities in the North-East of Germany (see Table 1 for more details). While *Ramalina* and *Cladonia* are rather common and widely distributed genera (Litterski, 1999), *Baeomyces* is less frequent (Wirth et al., 2013). The habitat of *Ramalina* is rather exposed to light, and it typically occurs among free-standing trees or building walls. In contrast, *Cladonia* prefers humid and shaded habitats, while *Baeomyces* can cope with both situations but is sensitive to eutrophication. After sampling, in order to avoid the dehydration of lichen thalli and to maintain their metabolic activity, thalli were placed in transparent plastic boxes, providing 100% air humidity through a water layer at the base. The algal cultures and the lichen specimens were incubated at 20°C and 30 μmol photons m^−2^s^−1^ in a light/dark cycle of 16:8 h for approximately 5 weeks.

### Cold and high light treatment experiment

**Figure 1:**
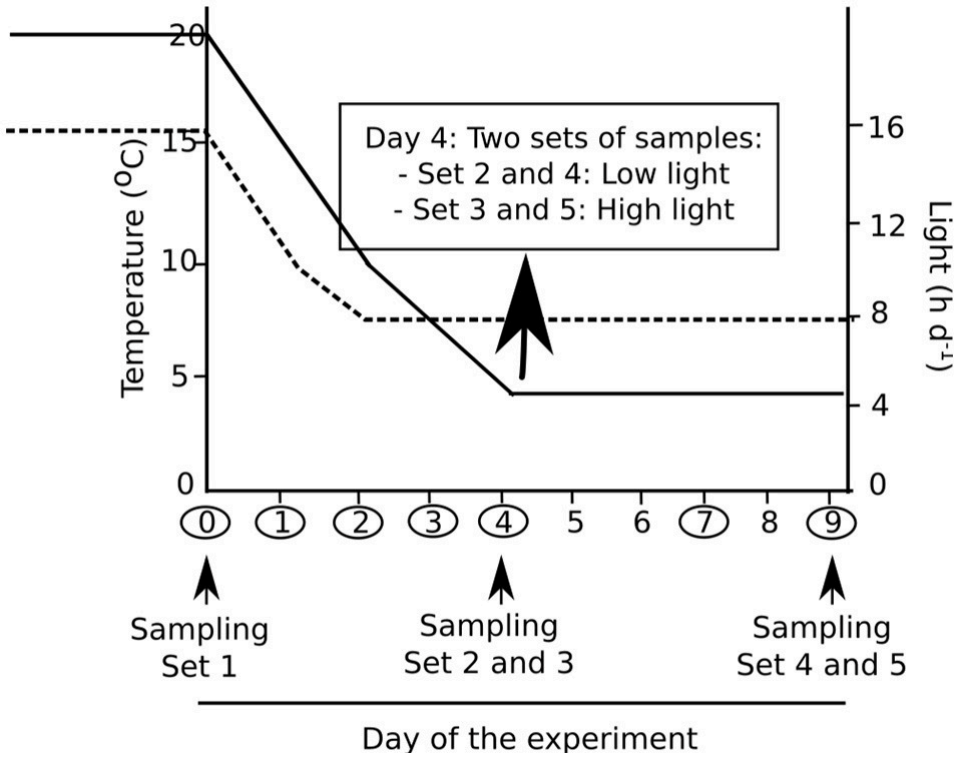
Experimental design of the cold acclimation treatment applied to algae and lichens. Temperature decreased from 20 to 5°C in 5 days; it was then maintained at 5°C during the next 5 days (solid line). Photoperiod decreased from 16 to 8 h (broken line). Photon flux density was 30 μmol photons m^−2^s^−1^ [low light (LL)] from the start of the experiment for all sets of samples, with the exception of sets 3 and 5, which were under 300 μmol photons m^−2^s^−1^ [high light (HL)] from day 4 to day 9. The days when fluorescence was recorded are indicated by circles, and samplings for pigment and low molecular weight carbohydrate analyses are indicated by arrows.

Prior to the cold treatment, algal cultures were transferred to sterile cell culture flasks (Corning, NY, USA; size 25 cm^2^) filled with 3N-BBM+V. The flasks were placed into incubators (Kunststoff-Technik Rostock, Rostock, Germany), which guaranteed constant temperatures (±0.1°C) and were equipped with LED arrays, ensuring an efficient regulation of the photon flux density (PFD). The transfer of the cell culture flasks inside the incubator was carried out 3 days prior to the start of the experiment to ensure acclimation to the given abiotic conditions (Gustavs et al., 2009). Lichen thalli maintained in the previously described plastic containers were placed in a modified wine storage cabinet (Liebherr, Biberach a der Riss, Germany), which was irradiated by a set of 6 Lumitronix LED strips. During the first 5 days of the experiment, temperature was down regulated from 20 to 5°C, while day length decreased in parallel from 16 to 8 h. The next 5 days, algae and lichens were maintained under cold and short-day conditions (5°C and 8 h of light), facing either a low light (LL: 30 μmol photons m^−2^s^−1^) or high light (HL: 300 μmol photons m^−2^s^−1^) treatment. The exact time course of temperature, irradiation and day length changes throughout the experiment is illustrated in Figure 1. Additionally, the dates of fluorescence measurements and samplings are indicated.

### Fluorescence analysis

Electron Transport Rate/Irradiance curves (ETR/I curves) were determined by a PAM-2500 fluorometer (Walz, Effeltrich, Germany). The algal cultures were filtered on glass fiber GF/F filter discs (diameter 25 mm, pore size 1–3 μm; Whatman GmbH, Dassel, Germany), which were dark adapted for 20–30 min. To avoid desiccation stress during the dark adaptation and measuring period, the filter with the algal sample was placed above another filter wetted with media. Then, they were exposed to a continuously increasing light gradient consisting of 13 steps of 1 min each. PFD ranged from 12 to 489 μmol photons m^−2^s^−1^. After each irradiation step, a saturating pulse was applied to obtain the maximum chlorophyll fluorescence (F_m_'). Along ETR/I curve measurements, the samples were maintained at the treatment temperature.

ETR, effective photochemical quantum yield of photosystem (Φ_PSII_), minimum chlorophyll fluorescence under irradiation (F_o_'), maximal photochemical efficiency of PSII (F_v_/F_m_) and non-photochemical quenching (NPQ) were calculated as follows:

(1) $$\text{ETR}=\text{PFD}\times \Phi_{\text{PSII}}$$

                                                       (According to Masojídek et al., 2001)

(2) $$\Phi_{\text{PSII}}=({\text{F}}_{\text{m}}^{\prime }-\text{F})/{\text{F}}_{\text{m}}^{\prime }$$

                                                       (According to Genty et al., 1989)

(3) $${\text{F}}_{\text{o}}^{\prime }={\text{F}}_{\text{m}}^{\prime }\times (1-\Phi_{\text{PSII}})$$

(4) $${\text{F}}_{\text{v}}/{\text{F}}_{\text{m}}=({\text{F}}_{\text{m}}-{\text{F}}_{\text{o}})/{\text{F}}_{\text{m}}$$

(5) $$\text{NPQ}={\text{F}}_{\text{m}}-{\text{F}}_{\text{m}}^{\prime }/{\text{F}}_{\text{m}}^{\prime }$$

ETR/I curves were fitted to the mathematical model of Platt et al. (1980):

(6) $$\begin{array}{l} \text{ETR}={\text{ETR}}_{\text{max}}\times (1-\text{exp}(-\alpha \times \text{PFD}/{\text{ETR}}_{\text{max}}))\\ \;\times \text{exp}(-\beta \times \text{PFD}/{\text{ETR}}_{\text{max}}) \end{array}$$

where ETR is the electron transport rate, ETR_max_ is the maximal electron transport rate, α is the photochemical efficiency (equivalent to the initial slope of the curve), PFD is the photon flux density and β is the term for photoinhibition.

### Sample preparation

Algal cells were harvested by two consecutive centrifugations for 5 min each at LT (5°C). The first centrifugation reduced the complete culture volume harvested from the cell culture flasks (5,000 g; Heraeus Megafuge 1.0 R, Heraeus GmbH, Dassel, Germany), while the second centrifugation allowed the partition of highly concentrated biomass in several aliquots for subsequent extractions (14,000 g; Heraeus Biofuge primo R, Heraeus GmbH, Dassel, Germany). After centrifugation, the pellets were frozen in liquid nitrogen and stored at −80°C until extraction. To obtain the dry weight of algal pellets, they were vacuum-evaporated in a Savant SpeedVac (SPD 111 V, ThermoFisher Scientific, Waltham, USA) connected to a Lyovac GT2 Freeze dryer (Steris, Cologne, Germany).

### Low molecular weight carbohydrate (LMWC) determination

Dry algal samples (7–12 mg dry weight) were extracted according to Karsten et al. (1991). After centrifugation for 5 min at 5,000 g, 700 μl of the supernatant were evaporated to dryness under vacuum (Speed Vac Concentrator SVC 100H, Savant). Dried extracts were dissolved again in 700 μl distilled water, vortexed for 30 s and treated in an ultrasonic bath for 5 min (Bandelin Sonorex, Berlin, Germany). After centrifugation, the clear supernatant was pipetted into high pressure liquid chromatography (HPLC) vials and closed with a membrane-equipped lid.

The detection of LMWCs was carried out with an Agilent HPLC system equipped with a refractive index detector (RID G1362A, Agilent, Santa Clara, USA) following two isocratic methods, depending on the investigated species. The determination of sucrose and ribitol was performed on a Bio-Rad resin-based column (Aminex Fast Carbohydrate Analysis, 100 × 7.8 mm) using a Phenomenex Carbo-Pb^2+^ (4 × 3 mm) guard cartridge. These carbohydrates were eluted with 100% HPLC-grade water at a flow rate of 1 ml min^−1^ at 70°C (Karsten et al., 1991). To separate various polyols from each other, LMWC analysis was performed on a Phenomenex Rezex ROA-Organic Acid resin-based column (300 × 7.8 mm) protected with a Phenomenex Carbo-H^+^ guard cartridge (4 × 3 mm). On the latter column, carbohydrates were eluted with 5 mM H_2_SO_4_ at a flow rate of 0.4 ml min^−1^ at 75°C (Karsten et al., 2005).

### Pigment analysis

The pigments analyzed in this work were: chlorophyll (Chl), lutein (L), antheraxanthin (A), zeaxanthin (Z), violaxanthin (V) and β-carotene (β-car). Dry algal samples were extracted twice. First, they were homogenized with a mortar in acetone (95%) buffered with CaCO_3_ (0.5 g l^−1^). Afterward, the extracts were centrifuged at 16,100 g for 5 min. Then, the pellet was resuspended in pure acetone buffered with CaCO_3_ with a Tissue Tearor Homogenizer (Model 395, Dremel, Mexico). The extract was centrifuged again at 16,100 g for 5 min, and both supernatants were mixed and were filtered with 0.2 μm PTFE filters (Tecknokroma, Barcelona, Spain). In the case of lichens, to avoid chlorophyll (Chl) degradation, the extraction was carried out using 95% acetone buffered with 0.5% NEDPA and the same amount of CaCO_3_ as the sample. Lichen extracts were centrifuged at 16,100 g and 4°C for 10 min, and supernatants were also filtered with 0.2 μm PTFE filters (Teknokroma, Spain). During the whole process, samples were maintained at a temperature approximately of 4°C to avoid pigment degradations. Pigment separation was performed by HPLC with a reversed-phase C18 column (Waters Spherisorb ODS1, 4.6 × 250 mm, Milford, MA, USA) with a photodiode array (PDA) detector, following the method of García-Plazaola and Becerril (1999), since modified by Garcia-Plazaola and Esteban (2012).

### Statistics

Kolmogorov–Smirnov and Cochran tests were used to test for the normality of data and homogeneity of variances, respectively. The *t*-student test was used for the calculation of differences between the NPQ (%) and F_*v*_/F_*m*_ (%). One-way ANOVA and Kruskal-Wallis tests were applied to check for differences in pigments and LMWCs in normal and non-normal data, respectively. Duncan and Dunnett *post hoc* tests were performed to discriminate changes in pigment content throughout the treatment in each species. In the case of non-normal data, Mann-Whitney U non-parametric tests were carried out. All analyses were performed using the SPSS 17.0 statistical package (SPSS, Armonk, NY, USA).

## Results

### Changes in photochemical efficiency, photosynthetic parameters and non-photochemical quenching during cold acclimation

**Figure 2:**
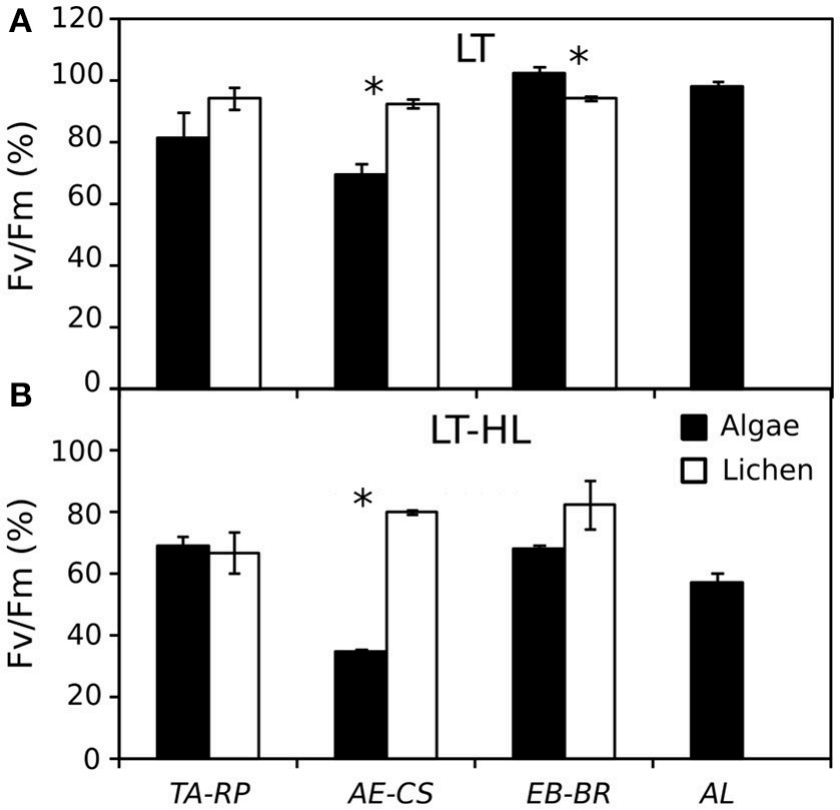
Percentage of F_v_/F_m_ (% F_v_/F_m_) at the end of the experiment with respect to the maximum value of the control replicates. **(A)** Values after 10 days of low temperature (LT). **(B)** Values after 4 days under the combination of LT with high light (LT-HL). Black and white bars represent algae and lichens, respectively. The average of Fv/Fm control values for each species were as follows: 0.619 for *TA*; 0.696 for *RP*; 0.565 for *AE*; 0.709 for *CS*; 0.560 for *EB*; 0.705 for *BR*; and 0.584 for *AL. AL, Apatococcus lobatus*; *EB, Elliptochloris bilobata*; *AE, Asterochloris erici*; *TA, Trebouxia arboricola*; *BR, Baeomyces rufus*; *CS, Cladonia squamosa*; *RP, Ramalina pollinaria*. Each bar represents the mean ± SE (*n* = 3). Asterisks indicate significant differences between the couples of species.

Photophysiological changes associated with the process of cold acclimation (CA) were monitored for 10 days in four isolates of terrestrial green algae (Chlorophyta, Trebouxiophyceae) and three lichens (Ascomycota, Lecanoromycetes, with trebouxiophyte photobionts) (Table 1). Changes in chlorophyll fluorescence showed a photoinhibitory effect from the LT-HL combination in all studied organisms but also revealed species-specific response patterns within the investigated couples of algal-lichen species. Thus, *TA* did not exhibit any significant difference with *RP* under LT or the combination of LT and HL. In contrast, *AE* showed significantly lower F_v_/F_m_ values than its respective lichen *CS* under both types of stresses. Surprisingly, the couple *EB*-*BR* showed the opposite pattern of response, with a higher photochemical efficiency in the free-living alga *EB* under stress (Figure 2).

**Figure 3:**
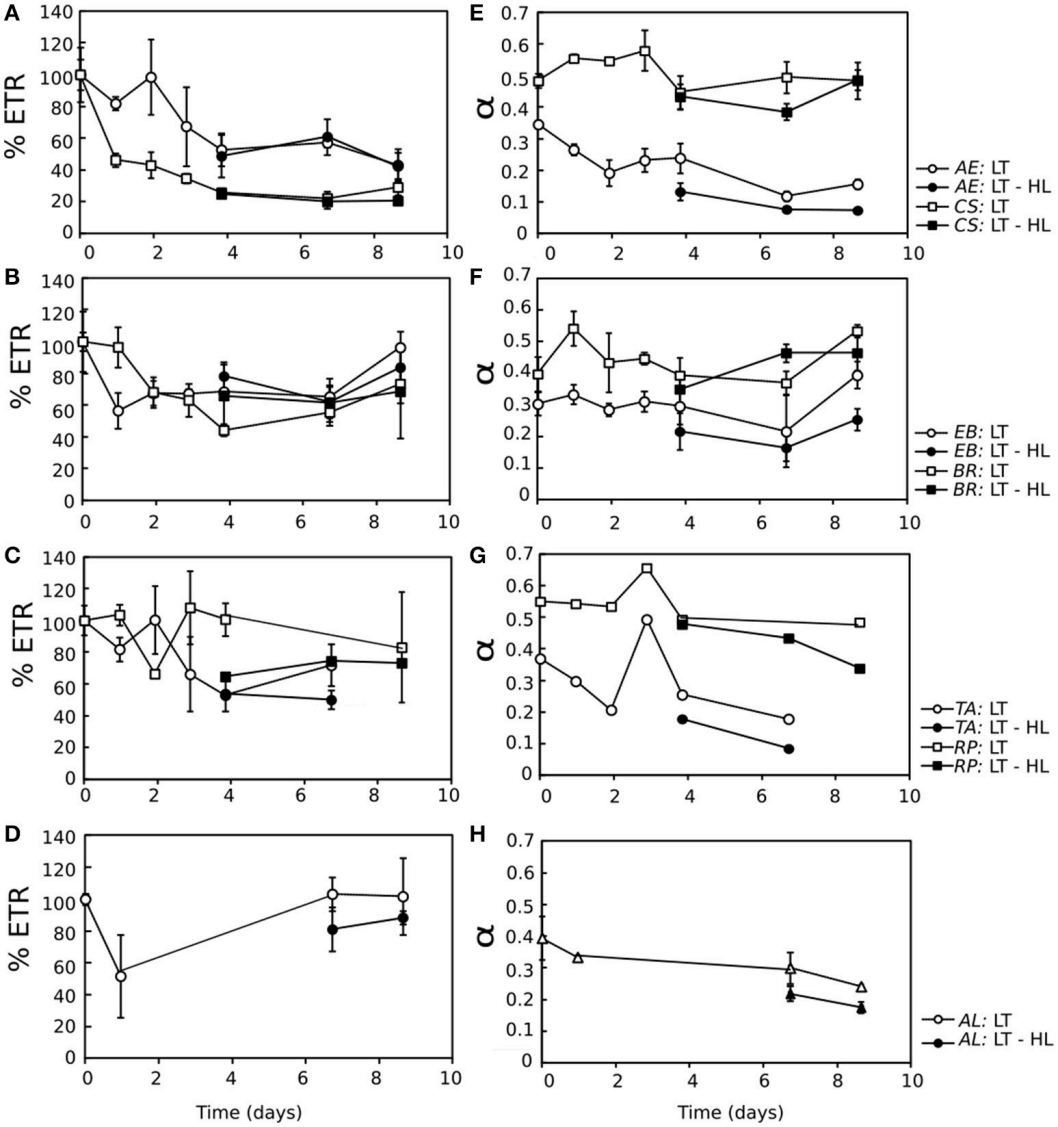
**(A–D)** Electron transport rate (ETR) derived from ETR/Irradiance curves, represented as the percentage with respect to the control value (% ETR) for all the studied species throughout the cold acclimation experiment (LT) (open symbols) and under the combination of low temperature with high light (LT-HL) (closed symbols). The average ETR control values for each species were as follows: 18.84 for *TA*; 37.31 for *RP*; 17.74 for *AE*; 99.84 for *CS*; 15.10 for *EB*; 32.11 for *BR*; and 10.74 for *AL*. **(E–H)** Photosynthetic efficiency (α) for all the studied species throughout the cold acclimation experiment (LT) (open symbols) and under the combination of low temperature with high light (LT-HL) (closed symbols). Circles indicate values for algae, while squares represent values for lichens. ETR and α were calculated using the Platt photosynthetic equation (Platt et al., 1980). *AL, Apatococcus lobatus*; *EB, Elliptochloris bilobata*; *AE, Asterochloris erici*; *TA, Trebouxia arboricola*; *BR, Baeomyces rufus*; *CS, Cladonia squamosa*; *RP, Ramalina pollinaria*. Each point represents the mean ± SE (*n* = 3). In the case of *AL*, some values are missing due to technical problems with the controlled temperature.

To analyze the time course of photosynthetic acclimation to LT and LT-HL in more detail, three photosynthetic parameters derived from ETR/I curves (Appendix S2) were followed throughout the stress treatments (Figure 3). ETR_max_ decreased in all species under LT exposure (Figures 3A–D), although it was slightly more pronounced in the algae. Nevertheless, after 2–4 days of CA, there was a trend of ETR_max_ stabilization (Figures 3A–D), with the exception of *AL*, in which ETR_max_ even recovered to the initial values at the end of the LT exposition (Figure 3D). In the case of α, this parameter was higher under LT stress compared to LT-HL in both lichens and algae during the acclimation process (Figures 3E–H). Although α diminished slightly during the CA process, it recovered to control values in all lichens and in the alga *EB*.

**Figure 4:**
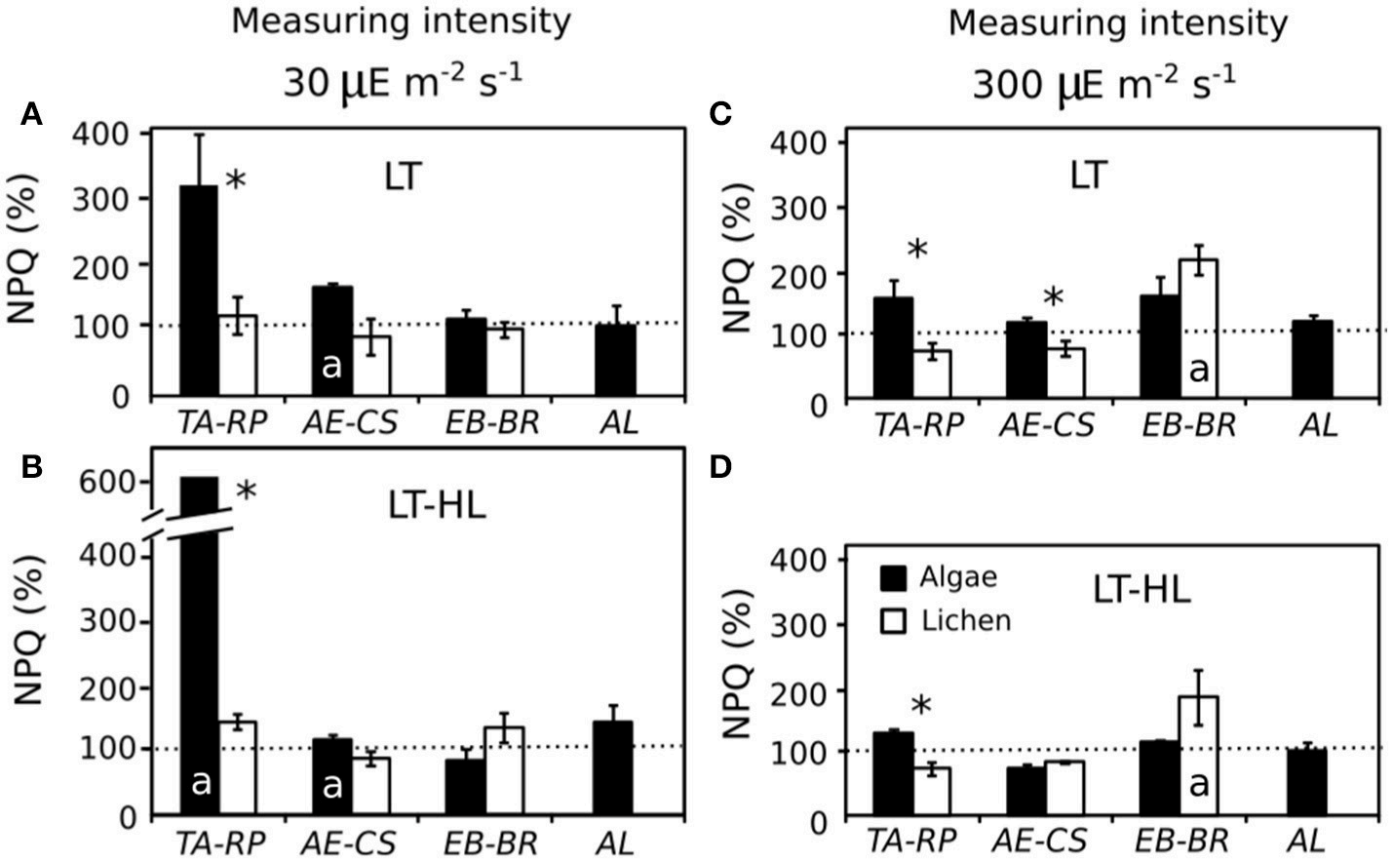
Percentage of NPQ (% NPQ) at the end of the experiment with respect to the average control values. These values were extracted from the light curves (Electron transport rate/Irradiance curves). **(A,B)** Values obtained at 30 μmol photons m^−2^s^−1^ for low temperature (LT) and low temperature with high light (LT-HL) treatments respectively. **(C,D)** Values obtained at 300 μmol photons m^−2^s^−1^ for LT and LT-HL treatments, respectively. Horizontal discontinuous line indicates 100%, which is equivalent to the initial value under control conditions. The average control NPQ values for each species at 30 μmol photons m^−2^s^−1^ were as follows: 0.16 for *TA*; 0.24 for *RP*; 1.00 for *AE*; 0.92 for *CS*; 1.43 for *EB*; 0.55 for *BR*; and 0.75 for *AL*. The average control NPQ values for each species at 300 μmol photons m^−2^s^−1^ were as follows: 1.90 for *TA*; 1.01 for *RP*; 1.93 for *AE*; 2.61 for *CS*; 2.95 for *EB*; 1.03 for *BR*; and 1.73 for *AL. AL, Apatococcus lobatus*; *EB, Elliptochloris bilobata*; *AE, Asterochloris erici*; *TA, Trebouxia arboricola*; *BR, Baeomyces rufus*; *CS, Cladonia squamosa*; *RP, Ramalina pollinaria*. Each bar represents the mean ± SE (*n* = 3). Asterisks indicate significant differences between the couples of species. Letter “a” inside the bars indicate that there are significant differences between NPQ value under stressed conditions (chilling and photochilling) and the control value.

In the continuously rising light gradient of each ETR/I curve, the calculation of NPQ was performed during the PFD acclimation of the experiment: 30 and 300 μmol photons m^−2^s^−1^ for LL and HL, respectively. The most dramatic increase in NPQ at both photon flux rates (with respect to the control values) was observed in *TA* (up to an increase of 500%) under LT-HL treatment (Figures 4A,B), while its lichen partner (*RP*) did not show such enhancement. This dramatic response was observed at 30 μmol photons m^−2^s^−1^ PFD in cultures acclimated in LT, and the response was even more pronounced in cultures acclimated in the combined LT-HL stress (Figures 4A,B, respectively). In contrast, in the remaining species, NPQ did not change as markedly as occurred in *TA*, and the percentage of NPQ did not differ in the other alga-lichen couples. When NPQ was measured at a PFD of 300 μmol photons m^−2^s^−1^ (Figures 4C,D), *TA* was also significantly higher than *RP* for both LT and HL-LT treatments, but the enhancement of NPQ was much smaller than at 30 μmol photons m^−2^s^−1^ PFD. The same occurred for the *AE*-*CS* couple but only under LT stress. Apart from comparing the two species of each couple, it was relevant to determine if the treatment had any effect in each species. The results showed that the species more affected by LT and HL-LT stresses were *AE* and *BR* under 30 and 300 μmol photons m^−2^s^−1^, respectively (Figure 4).

### Biochemical response under HL-LT treatment

**Figure 5:**
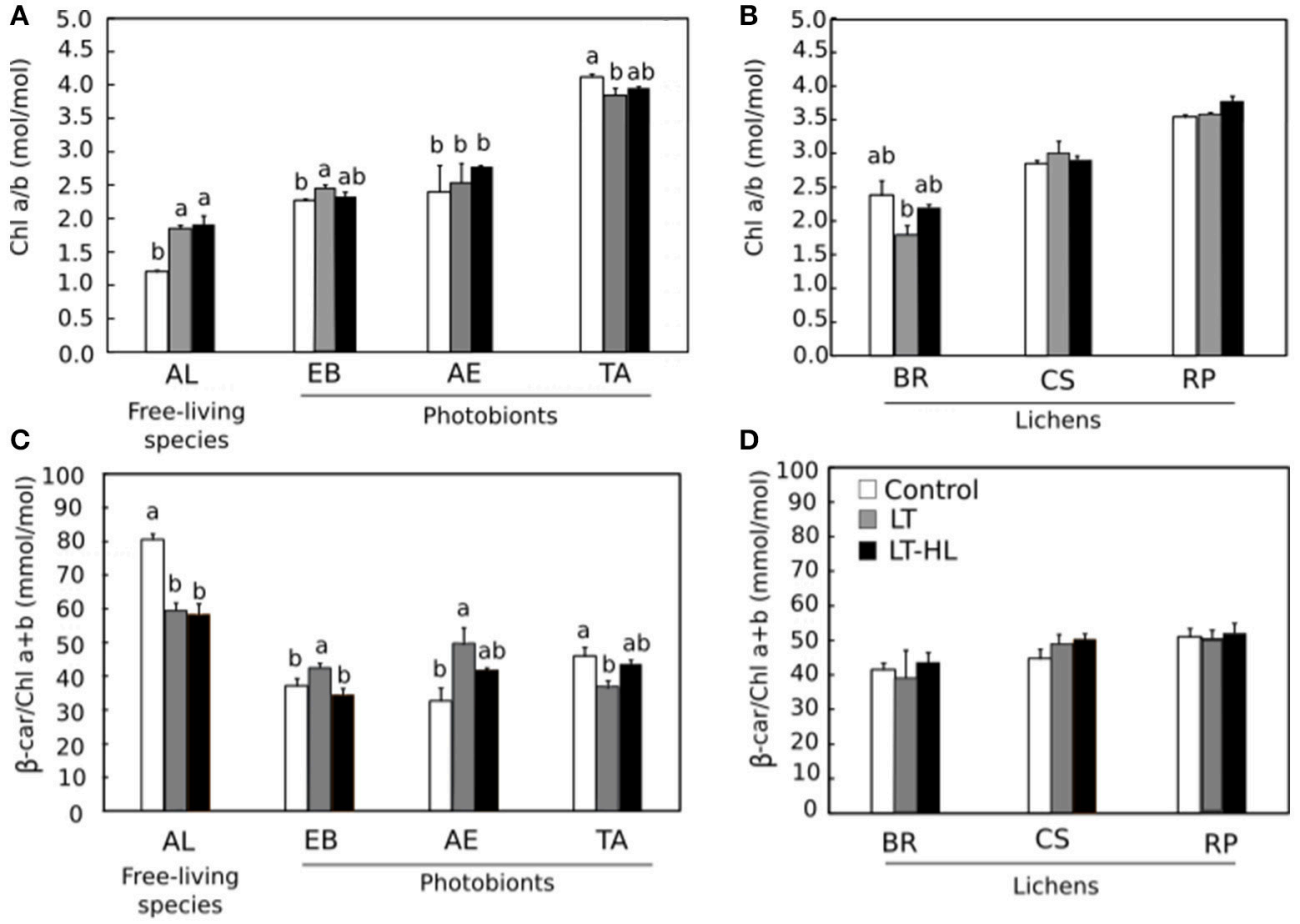
Effects of experimental treatments on pigment composition in free algae (left panels) and lichens (right panels). *AL, Apatococcus lobatus*; *EB, Elliptochloris bilobata*; *AE, Asterochloris erici*; *TA, Trebouxia arboricola*; *BR, Baeomyces rufus; CS, Cladonia squamosa*; *RP, Ramalina pollinaria*. Panels **(A,B)** show Chl a/b ratios in algae and lichens respectively while **(C,D)** show β-car/Chla+b ratios in algae and lichens respectively. White color indicates control values under 20°C. Gray bars refer to LT (low temperature) treatment and black bars refer to LT-HL (low temperature-high light) treatment. Data are mean ± SE (*n* = 3). Letters indicate significant differences for each species at *P* < 0.05. The absence of letters means no significant differences.

**Figure 6:**
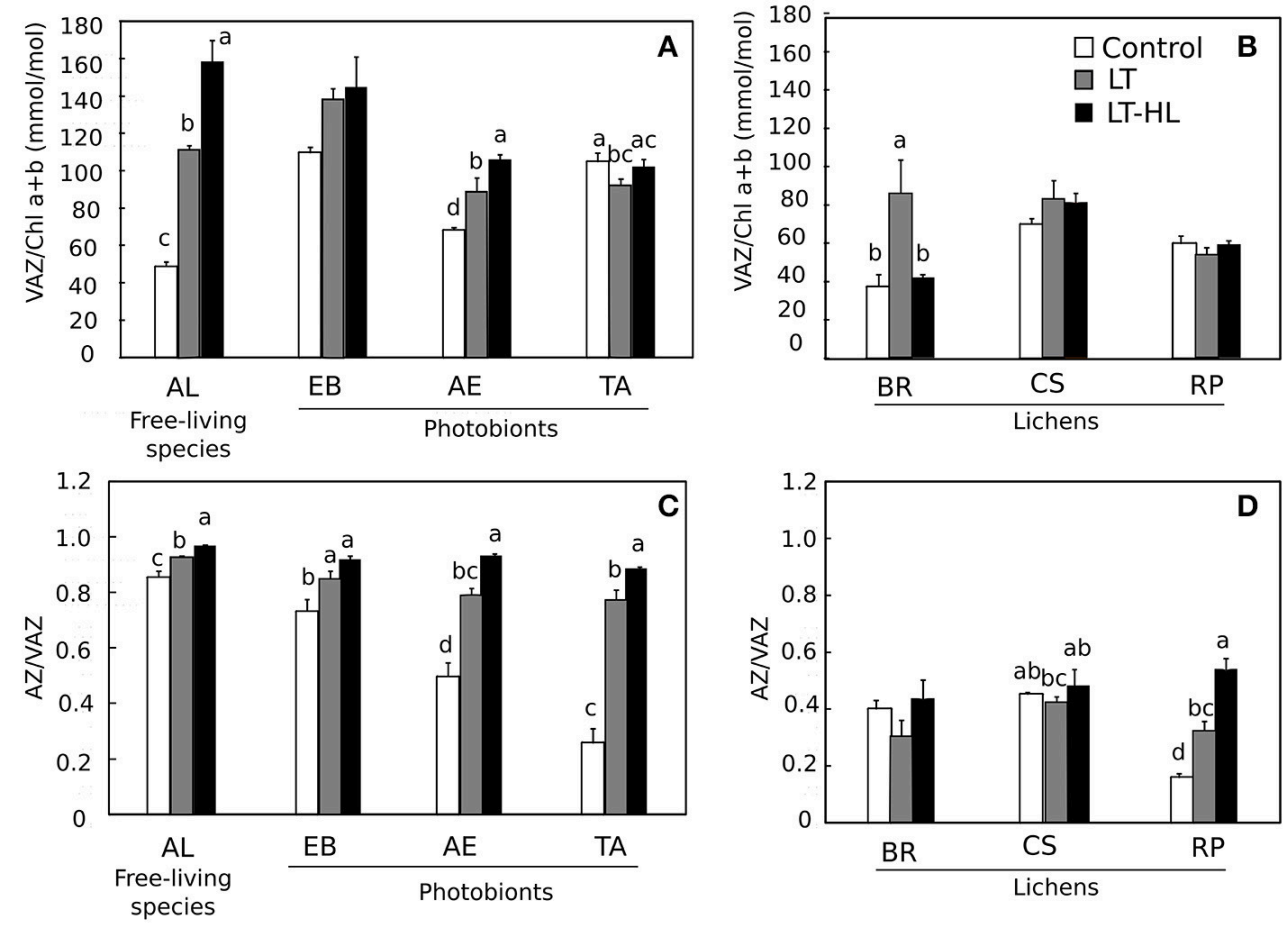
Effects of experimental treatments on violaxanthin cycle (V-cycle) components in free algae (left panels) and lichens (right panels). V, violaxanthin; A, anteraxanthin; Z, zeaxanthin. *AL, Apatococcuslobatus*; *EB, Elliptochlorisbilobata*; *AE, Asterochloriserici*; *TA, Trebouxiaarboricola*; *BR, Baeomycesrufus; CS, Cladoniasquamosa*; *RP, Ramalinapollinaria*. Panels **(A,B)** show VAZ/Chla+b in algae and lichens respectively while panels **(C,D)** show AZ/VAZ in algae and lichens respectively. White color indicates control values under 20°C. Gray bars refer to LT (low temperature) treatment and black color refers to LT-HL (low temperature-high light) treatment. Data are mean ± SE (*n* = 3). Letters indicate significant differences for each species at *P* < 0.05. The absence of letters means no significant differences.

**Figure 7:**
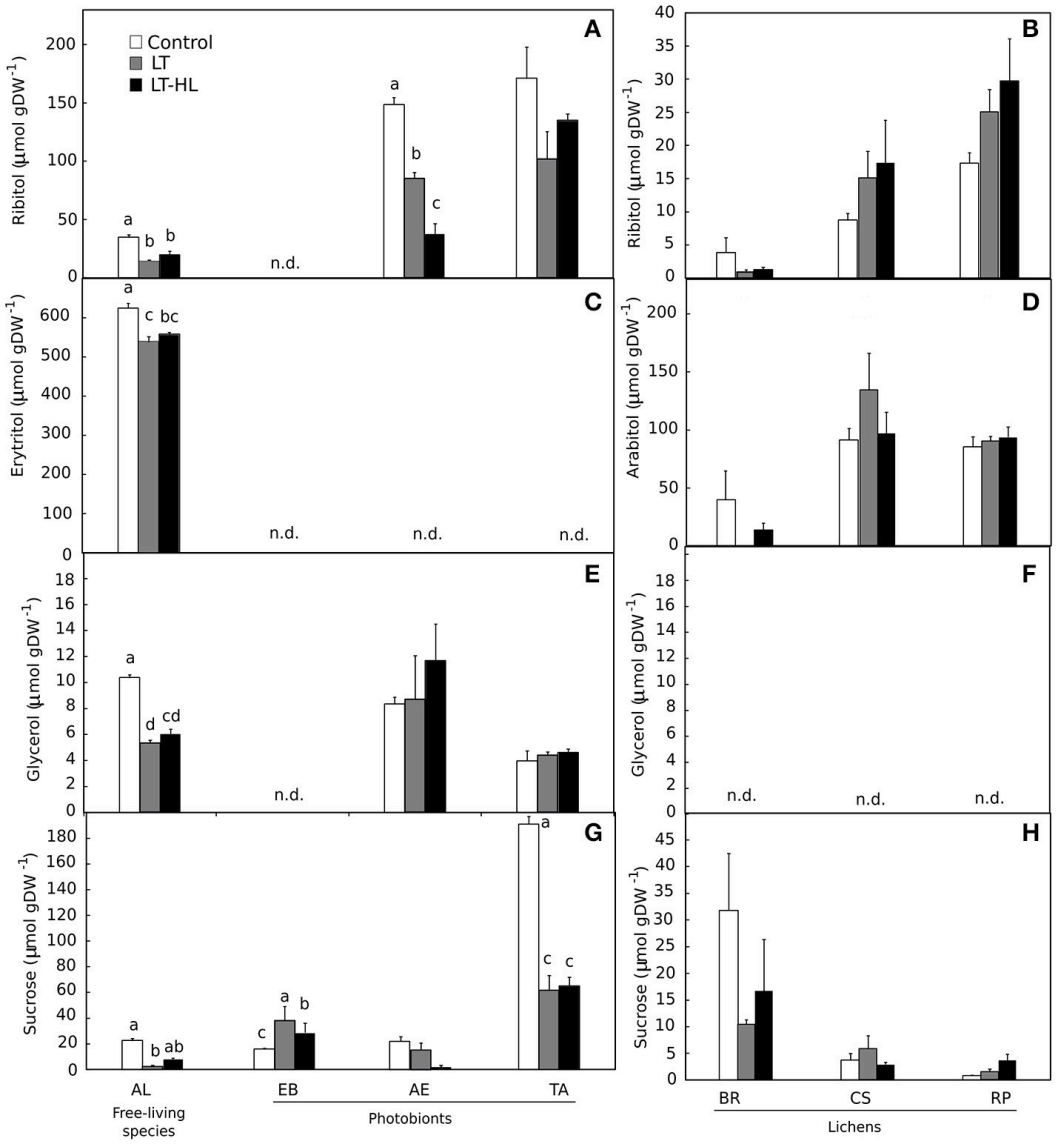
Effects of experimental treatments on low molecular weight carbohydrates (LMWC) in free algae (left panels) and lichens (right panels). *AL, Apatococcus lobatus*; *EB, Elliptochloris bilobata*; *AE, Asterochloris erici*; *TA, Trebouxia arboricola*; *BR, Baeomyces rufus; CS, Cladonia squamosa*; *RP, Ramalina pollinaria*. Panels **(A,B)** show ribitol content in algae and lichens respectively; panels **(C,D)** show erytritol content for algae and arabitol content for lichens respectively; panels **(E,F)** show glycerol content in algae and lichens respectively, and panels **(G,H)** show sucrose content in algae and lichens respectively. White color indicates control values under 20°C. Gray bars refer to LT (low temperature) treatment and black colors refer to LT-HL (low temperature-high light) treatment. All LMWC concentrations are given as μmol g^−1^ dry weight (DW). Data are mean ± SE (*n* = 3). Letters indicate significant differences for each species at *P* < 0.05. The absence of letters means no significant differences.

To understand the biochemical mechanisms underlying the photochemical responses associated with CA process itself, and in combination with HL stress, the pools of pigments and low molecular weight carbohydrates (LMWCs) were quantified during the stress treatments (Figures 5–**7**, respectively). The respective figures display the variation between control and final value (to see the complete pigment and LMWC datasets, see Appendix S3). Most relevant changes of photosynthetic pigments during the temperature and light treatments included the Chl a/b ratio increasing significantly in *AL* (Figure 5A), while the β-car showed the opposite pattern in the same species (Figure 5C). In the other algal species and the entire set of lichen specimens, these two pigments remained unchanged during the course of the experiment. The V-cycle pigment pool (VAZ/Chl) increased from LT and LT-HL exposure in most algal species (*AL, EB, AE*) (Figure 6A) and in the lichen *BR* (Figure 6B). The de-epoxidation state of xanthophyll cycle pigments (AZ/VAZ) also increased, not only under the combined LT-HL treatment but also under LT stress in all species, except *BR* and *CS*. These increases were more pronounced when LT was combined with HL compared to LT alone (Figures 6C,D).

The basic composition of LMWCs (ribitol, erythritol, arabitol, glycerol and sucrose) differed among species (Figure 7). Sucrose was ubiquitous and ribitol was present in all species except *EB*, while the presence of other LMWCs analyzed were taxonomically more restricted. The patterns of response to the experimental treatment can be basically described as a net decrease or maintenance of the pools of these compounds during the stress period. The only noteworthy exception was that *EB* showed an increase in sucrose content for both the LT and the combination of LT and HL treatments (Figure 7G).

## Discussion

In the present study, we investigated the physiological responses to chilling and photochilling stresses of three lichen specimens (*RP, CS*, and *BR*) associated with different trebouxiophyte photobionts growing in different light-influenced habitats in northern Germany. The typical photobiont species (*TA, AE*, and *EB*) associated with those target lichens, as well as one free-living terrestrial alga (*AL*), were analyzed as monoclonal cultures under the same stress conditions. It was not possible to isolate the photobionts from the investigated lichen thalli, but photobionts were accessed from the most suitable/compatible unialgal strains, available from a public culture collection (SAG – Sammlung von Algenkulturen, Göttingen, Germany) (Table 1). Therefore, they are considered here as “associated photobionts,” and the three algal strains have all been originally isolated from lichen thalli native to areas with a strong winter season. Therefore, all photobionts studied here are naturally exposed to stresses associated with LT.

This study has not been designed to systematically evaluate the role of phylogenetic position of the target genera in the overall photoprotective response. Addressing this task would have required a significantly enhanced number of algal strains, testing at least three strains per genus, or even better per species. Nevertheless, some general considerations based on literature can be summarized: the Trebouxiales clade contains currently three known genera, namely *Trebouxia, Asterochloris*, and *Myrmecia*, all are common and worldwide-distributed photobionts in lichen symbiosis (Friedl and Büdel, 2008; Friedl and Rybalka, 2012). *Elliptochloris* belongs to the sister clade of the Trebouxiales, the Elliptochloris-clade, which also comprises the genome sequenced model algae *Coccomyxa* and the biotechnological explored *Botryococcus* (Darienko et al., 2016). In contrast to *Trebouxia* and relatives, *Elliptochloris* and relatives occur in rather few lichen species (Gustavs et al., 2017 and references therein). The genus *Elliptochloris* exhibits a particular versatility in lifestyles from terrestrial to aquatic habitats and different symbiotic associations. The genus *Apatococcus* forms a distinct clade within the Trebouxiophyceae and is phylogenetically more distant to the further mentioned genera. It is considered the most common terrestrial green algae in temperate latitudes (Barkman, 1969; Gustavs et al., 2016; Hallmann et al., 2016). There is no literature available about the photochilling response of the genus *Apatococcus*, as its application in physiological or biochemical studies has long been hampered by an extraordinary poor growth performance under standard culture conditions (Darienko et al., 2015). In general, the literature on polar algae and lichen is much more extensive as they play a fundamental role in cold ecosystems where they thrive in harsh environments higher plants cannot colonize (Colesie et al., 2014).

### Sugar accumulation and the V-cycle: two mechanisms to compensate for (photo)chilling stress

Photosynthesis is not a continuous process, since it changes depending on environmental conditions. It is generally accepted that in lichens, photosynthetically produced sugars in algae are transferred to the mycobiont in form of ribitol (Lines et al., 1989). However, sugar formation and transference rates differ depending on the environmental conditions (light and temperature, among others). In this study, sugars tend to decrease over time, indicating that under (photo)chilling, photosynthesis rates are lower, or that the degradation of sugar is higher. The decrease of Fv/Fm in the present study demonstrated that the most plausible explanation is a reduction in photosynthesis activity. Colesie et al. (2014) revealed that the light compensation point, i.e., the photon flow rate where the rate of photosynthesis exactly matches the rate of respiration, depends on both the previously experienced light and temperature history of the respective organism. So, we could deduce that under low temperature, also the compensation points are lower and there are not necessary high light conditions due to the downregulation of metabolism.

Under LT, the CA process induces numerous physiological changes in photosynthetic organisms. The accumulation of sugars and the de-epoxidation of V-cycle pigments, as measured in this work, have been proposed to be relevant (e.g., Nagao et al., 2008; Adams et al., 2013; Trischuk et al., 2014), as both are involved in the regulation of the NPQ process. In the case of LMWCs, a recently postulated hypothesis considers some of these sugars to play an important role in NPQ under desiccation stress in *Trebouxia* sp. (Kosugi et al., 2013). These authors highlighted the importance of arabitol in NPQ formation, since this polyol acts as a modulator of gene expression and can change the conformation of proteins involved in photosynthesis during dehydration. Aside from this role, LMWCs contribute to freezing tolerance, acting as organic osmolytes, compatible solutes and cryoprotectants as well as signaling molecules (Janská et al., 2010; Theocharis et al., 2012). In the present work, sucrose content in *EB* increased after LT treatments (Figure 7G), while in the remaining species the accumulation of sugars was not a response to chilling and photochilling stresses. Nevertheless, in *EB*, NPQ did not increase under both stresses (Figure 4C); therefore, sugar content and NPQ were apparently not related. In contrast to *EB*, in the other free-living algae photochemical efficiency decreased after stress; in parallel, not only sucrose did decline but also concentrations of other LMWCs, such as ribitol, erythritol and glycerol. This response was previously described in different genotypes of two herbaceous angiosperms subjected to a CA period of 3 weeks at 2°C (Pociecha et al., 2010). The decline in LMWCs under LT and HL stress, as observed in most of the algal and lichen species, can be explained with an increase in fructan biosynthesis, as was reported by Pociecha et al. (2010). These authors determined that frost-resistant herbs accumulate mono- and disaccharides, while cultivars resistant to snow accumulated fructans. Hence, different biochemical patterns were described depending on the specific stress.

The V-cycle is one of the main components of the NPQ mechanism. Recent models proposed by Holzwarth et al. (2009) and Jahns and Holzwarth (2012) suggested the existence of two quenching sites. The quenching site known as Q1, which is located in the major light harvesting complex, does not require zeaxanthin (Z) for its activation, but it is amplified by the presence of this pigment. In addition, the Q2 quenching site, which is strictly Z-dependent, is located in the minor antennae proteins. This model, originally based on observations of higher plants, has also been used to understand the diversity of NPQ mechanisms reported in algae (Goss and Lepetit, 2015). Quaas et al. (2015) evaluated these two processes in terms of the dependence between NPQ and Z in a study about the activation of NPQ under HL in 6 different green algae. Based on their observations, these authors gave mechanistic support to the high diversity of NPQ mechanisms in algae. In agreement with such data, our results showed that in all algal species as well as in *RP*, there was an increase of the AZ/VAZ ratio during the treatment, but this index was not necessarily related to an increase in NPQ. In fact, both parameters (NPQ and AZ/VAZ) are only correlated in the *TA-RP* couple (Appendix S4).

Contrasting with most conifers where the accumulation of sugars and Z appear as complementary physiological adaptations in response to chilling and photochilling stresses (Verhoeven, 2014), in the present study, all the green algal species, with the exception of *EB*, accumulated Z but not sugars under LT and LT-HL stresses.

### Phylogeny or environmental pressure: what is determinant in physiological performance under (photo)chilling stress?

Both the environment and the phylogenetic position can influence the selection pressure of algal diversification. Taking all the results into consideration, the present study suggests that the environmental pressure is higher than phylogenetic constraints in terms of physiological adaptation under chilling and photochilling stresses and thus confirms reports of Quaas et al. (2015). Our conclusion is based on several observations. First, *EB* isolated from an alpine ecosystem (Table 1) (Eliáš et al., 2008) exhibited the best physiological performance under chilling stress compared to the other species. *EB* did not suffer any decrease of photochemical efficiency after the LT treatment; instead, it showed higher photochemical efficiency than its lichen partner *BR* after the chilling stress (Figure 2A), all presumably due to the sucrose accumulation. Second, *TA* exhibited a different response pattern, with strongly increasing NPQ, but not in the sugar content under chilling and photochilling stresses. *TA* is the dominant photobiont in continental Antarctic macrolichens (Helms et al., 2001), and under such extreme conditions, the increase of NPQ is of particular importance for avoiding damage in the photosynthetic apparatus.

The same response detected for *TA* was also observed for *AE*, which is phylogenetically closely related to *TA* (Helms et al., 2001; Piercey-Normore and Depriest, 2001). These data indicate that some physiological traits favorable for compensation of stress can also be phylogenetically preserved. The enhancement of NPQ observed in *TA* and *AE* under stress has also been described in other algal species under HL (Alou-Font et al., 2013; Katayama and Taguchi, 2013) and LT (Mock and Hoch, 2005), reflecting thermal dissipation as a widespread mechanism to compensate for both chilling and photochilling stresses.

### Algae: living isolated or in symbiosis with a fungus

Aero-terrestrial environments induced taxonomical widespread. As a consequence, different morphotypes and growth forms appeared during the algal evolution. These adaptations can induce identical morphology in unrelated evolutionary lineages (Rindi, 2011). In contrast to the algal species mentioned in the previous section (*TA* and *EB*), *AL* is never protected from the external environment by a fungal layer (Voytsekhovich, 2013). This may suggest it is not well adapted to aerial stresses. Nevertheless, it has been described very recently that it is the most dominant in algal covers on artificial hard substrate surfaces (Hallmann et al., 2016). At the same time, our results demonstrated that this species maintains the electron transport rate under both chilling and photochilling stresses. This success in subaerial habitats is the result of some specific adaptations. This alga grows in three-dimensional sarcinoid colonies (Appendix S1), sometimes forming thick cell walls and mucilage layers (Darienko et al., 2015; Hallmann et al., 2016). In addition, it has been observed that *Apatococcus* colonies present several layers of empty cells, covering the live cells (Hallmann et al., 2016). Previous studies on the terrestrial green algal genus *Interfilum* indicated that single cells, when they are strongly associated with other algal cells in an aggregate, colony or biofilm, are better protected against desiccation and other environmental stresses (Karsten et al., 2014). The formation of algal cell pockets could be related to a joint matrix of extracellular polysaccharides (EPS; mucilage), in which the individual cells are embedded. A study on freshwater *Coleochaete* species under simulated terrestrial culture conditions clearly indicated a strong change in morphology from typical radial thalli to the formation of packet-like structures (Graham et al., 2012). Therefore, the formation of a three-dimensional sarcinoid colony might be a morphological adaptation of free-living non-lichenized algae to thrive under terrestrial conditions. In agreement with this model, *AL* showed the highest amount of lipophilic antioxidants (tocopherols and β-car) during all the experimental treatments, even under initial control conditions (Figure 5 and Appendix S3). Antioxidants act as efficient protectors of thylakoids (Baroli and Niyogi, 2000). The accumulation of these protective compounds may help to maintain the balance among efficient light harvesting, photochemistry and photoprotection in *AL* biofilms.

The photobionts have often been considered the more sensitive partner within the lichen symbiosis (De Vera and Ott, 2010). Nevertheless, Sadowsky and Ott (2015) concluded from their recent study on Antarctic lichen, that the successful adaptation of lichens to the harsh natural conditions is in part based on the physiological potential of the photobionts. They investigated photosynthetic performance of the Antarctic foliose lichen *Umbilicaria decussata*. While the isolated *Trebouxia* photobionts displayed a clearly temperature dependent photoinhibition, no photoinhibition has been observed in the entire lichen thallus. Though the isolated photobiont is capable of excess light protection, the results clearly show that photoprotection is significantly increased in the symbiotic state, probably due to melanin synthesis. The closely related photobiont of *Pleopsidium chlorophanum*, a lichen lacking melanin, showed a higher potential of carotenoid-based excess light tolerance. This fact discriminates the two photobionts of the same *Trebouxia* clade. This example documents the tremendous physiological plasticity of the here investigated Trebouxiophyceae and underlines the initial statement that general conclusions can hardly been drawn. One of the main benefits that algae obtain from the lichen symbiosis is the light shielding caused by the accumulation of fungal secondary metabolites in the upper surface of the cortex (Lawrey, 1986). Nevertheless, the interactions between both organisms go beyond this passive protection, as many metabolic exchange processes exist, such as the accumulation of antioxidants and the activation of photoprotective mechanisms under stresses such as desiccation (Kranner et al., 2005). On the other hand, Sadowsky and Ott (2012) demonstrated that at freezing temperatures, *Trebouxia* individuals isolated from Arctic lichens are robust *per se* without the presence of the fungus. In the present study, two of the three photobionts in their free-living state did not show a lower photochemical efficiency with respect to their symbiotic state after the cold stress treatment. In addition, *EB* exhibited even higher values of photochemical efficiency than the respective lichen, *BR*. This reinforces the idea that the fungus is not essential for the survival of the photobiont under several environmental stresses. Therefore, we reject the *ad hoc* hypothesis that lichens show higher efficiency than free-living algae under photochilling stress.

## Conclusion

The present study was designed to evaluate exemplary and, for the first time, the (photo)chilling response of Trebouxiophyceae, which represents an ecologically diverse group of green algae. The algal genera *Asterochloris* (Škaloud et al., 2015), *Elliptochloris* (Darienko et al., 2016; Gustavs et al., 2017), *Trebouxia* (Seckbach et al., 2017) and *Apatococcus* (Hallmann et al., 2016) are considered ecologically and phylogenetically diverse. Recent publications exist for each of them, stating a general undersampling of these ecologically important microalgae. The investigated genera consist of various species exhibiting a set of different lifestyles, and a general conclusion regarding genus-specific patterns cannot be drawn from a study addressing one representative per genus.

**Figure 8:**
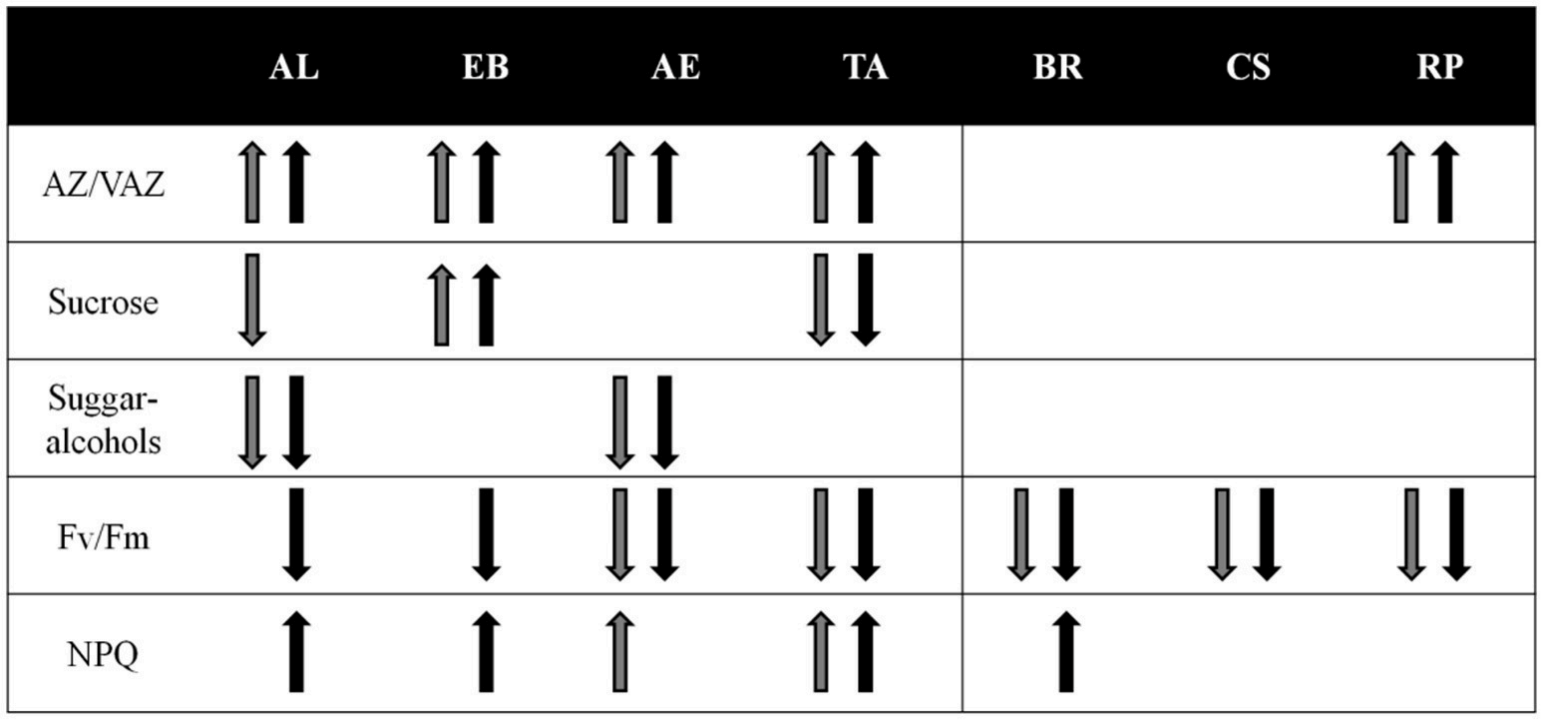
Summary of the physiological and biochemical responses of studied species during the experimental cold acclimation period. Gray and black arrows represent the trends of each parameter under low temperature and low (30 μmol photons m^−2^ s^−1^) high light (300 μmol photons m^−2^s^−1^) respectively (see Figure 1). When no arrows trends are shown, the parameter remained stable along the experimental treatment.

The variety of investigated processes across the investigated taxa obtained in the present study is illustrated in Figure 8. It summarizes the regulation potential of each species under (photo)chilling stresses. It also indicates that “lichenization matters,” but this is not a requisite for algae survival under harsh environmental conditions. The up- or down-regulation of physiological and biochemical parameters, as indicated by the arrows (Figure 8), is only occasionally found in the photobionts, while algal isolates regulate the majority of investigated traits. In addition, the data presented point to the environmental selection pressure as a more relevant factor than the phylogenetic position in the responses to environmental stresses; however, a broader dataset would be necessary to draw general conclusions.

## Author contributions

LG was the main investigator and supervisor of FM. US designed and participated in field expedition, determining sampling sites and collecting, identifying and maintaining the lichen species. FM and LG participated in samplings, as well as in the design of the photoinhibition experiment in growth chambers. Also they performed fluorescence measurements. LG determined sugar contents. FM determined pigment content, analyzed all the results and wrote the first draft of the manuscript. UK and JG participated in the interpretation of the results, as well as in the preparation and editing of the manuscript. All authors contributed to the final version of the manuscript.

### Conflict of interest statement

The authors declare that the research was conducted in the absence of any commercial or financial relationships that could be construed as a potential conflict of interest.

## Abbreviations

A: antheraxanthin
*AE*: *Asterochloris erici*
*AL*: *Apatococcus lobatus*
BBM: Bold's basal medium
*BR*: *Baeomyces rufus*
CA: cold acclimation
Chl: chlorophyll
*CS*: *Cladonia squamosa*
*EB*: *E. bilobata*
ETR: electron transport rate
F_*o*_': minimum chlorophyll fluorescence under illumination
F_*v*_/F_*m*_: maximal photochemical efficiency of PSII
F_*m*_': maximum chlorophyll fluorescence
HL: high light
HPLC: high pressure liquid chromatography
LL: low light
LMWC: low molecular weight carbohydrate
LT: low temperature
NPQ: non-photochemical quenching
PDA: photodiode array
PFD: photon flux density
ETR: maximum photosynthesis
PS: photosystem
ROS: reactive oxygen species
*RP*: *R. pollinaria*
*TA*: *T. arboricola*
TOM: Trebouxia organic media
V: violaxanthin
V-cycle: violaxanthin cycle
Z: zeaxanthin
α: photochemical efficiency
β: photoinhibition
Φ_PSII_: effective photochemical quantum yield of photosystem.
