# Supplementary material for: Unraveling the Photoprotective Response of Lichenized and Free-Living Green Algae (Trebouxiophyceae, Chlorophyta) to Photochilling Stress
Source: Front Plant Sci. 2017 Jul 4;8:1144. doi: 10.3389/fpls.2017.01144 (PMC5495867; doi:10.3389/fpls.2017.01144)

## APPENDIX S1

*Elliptochloris bilobata, Apatococcus lobatus, Asterochloris erici* and *Trebouxia arboricola*, under control conditions and after the 10 days of the experiment (5 days of cold acclimation and 5 days under low temperature conditions (lt) and low light (ll) or (hl)).

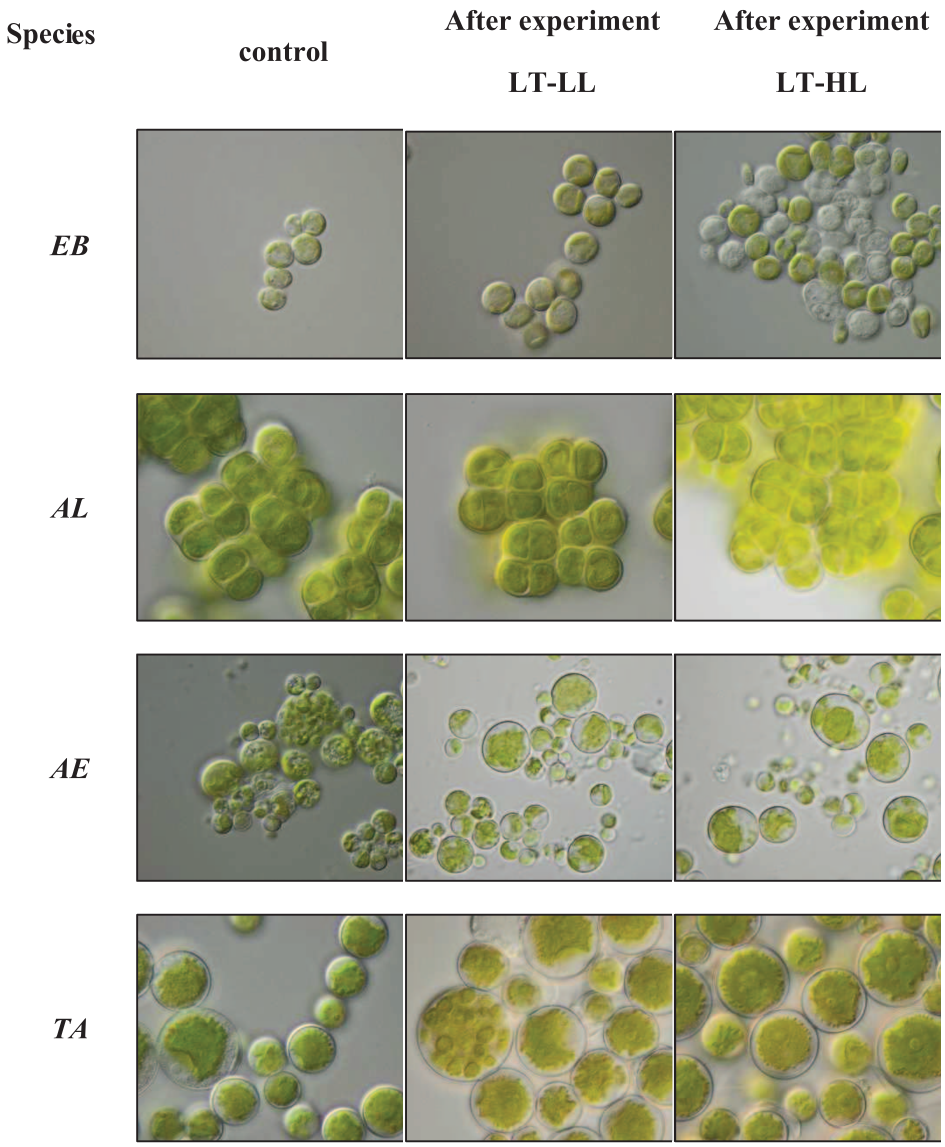

Supplement: Supplementary file 1 [file Presentation1.PDF]
