# Supplementary material for: Unraveling the Photoprotective Response of Lichenized and Free-Living Green Algae (Trebouxiophyceae, Chlorophyta) to Photochilling Stress
Source: Front Plant Sci. 2017 Jul 4;8:1144. doi: 10.3389/fpls.2017.01144 (PMC5495867; doi:10.3389/fpls.2017.01144)

Appendix S2: Electron transport rate/irradiance curves (ETR/I curves) for each alga and lichen species. In each graph, three replicates for each day of measurement were represented.

Cladonia squamosa

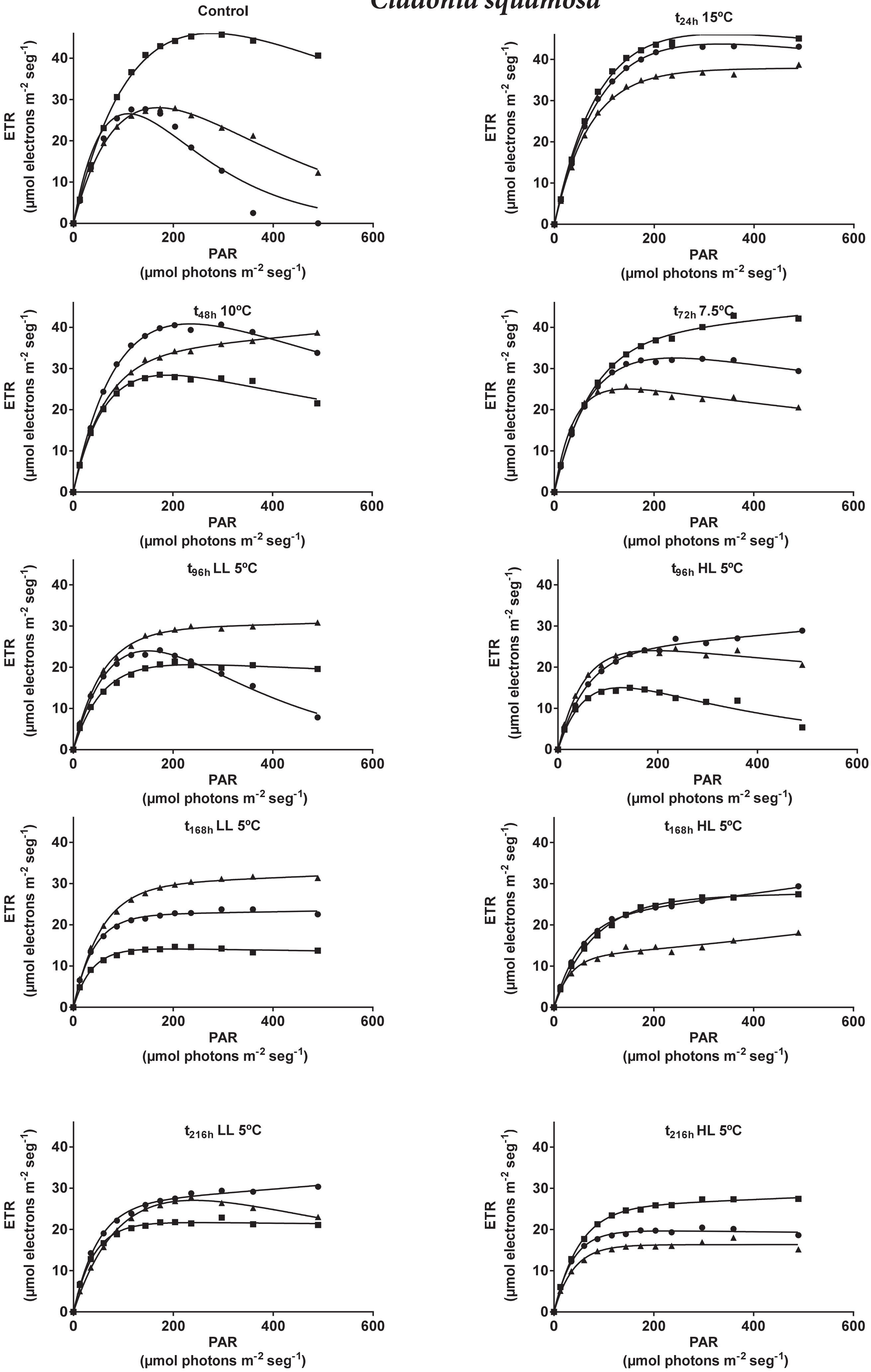

Baeomyces rufus

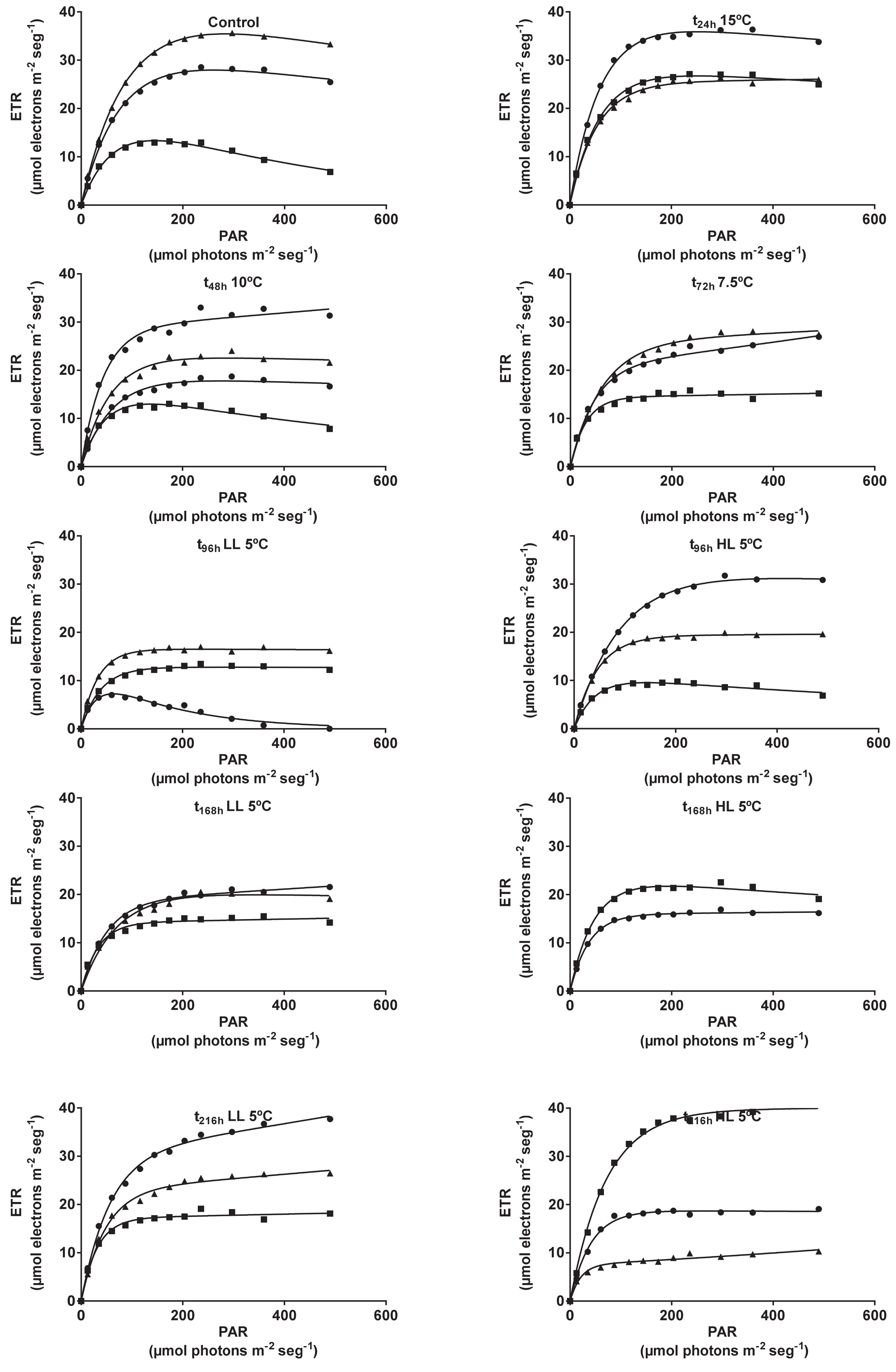

Asterochloris erici

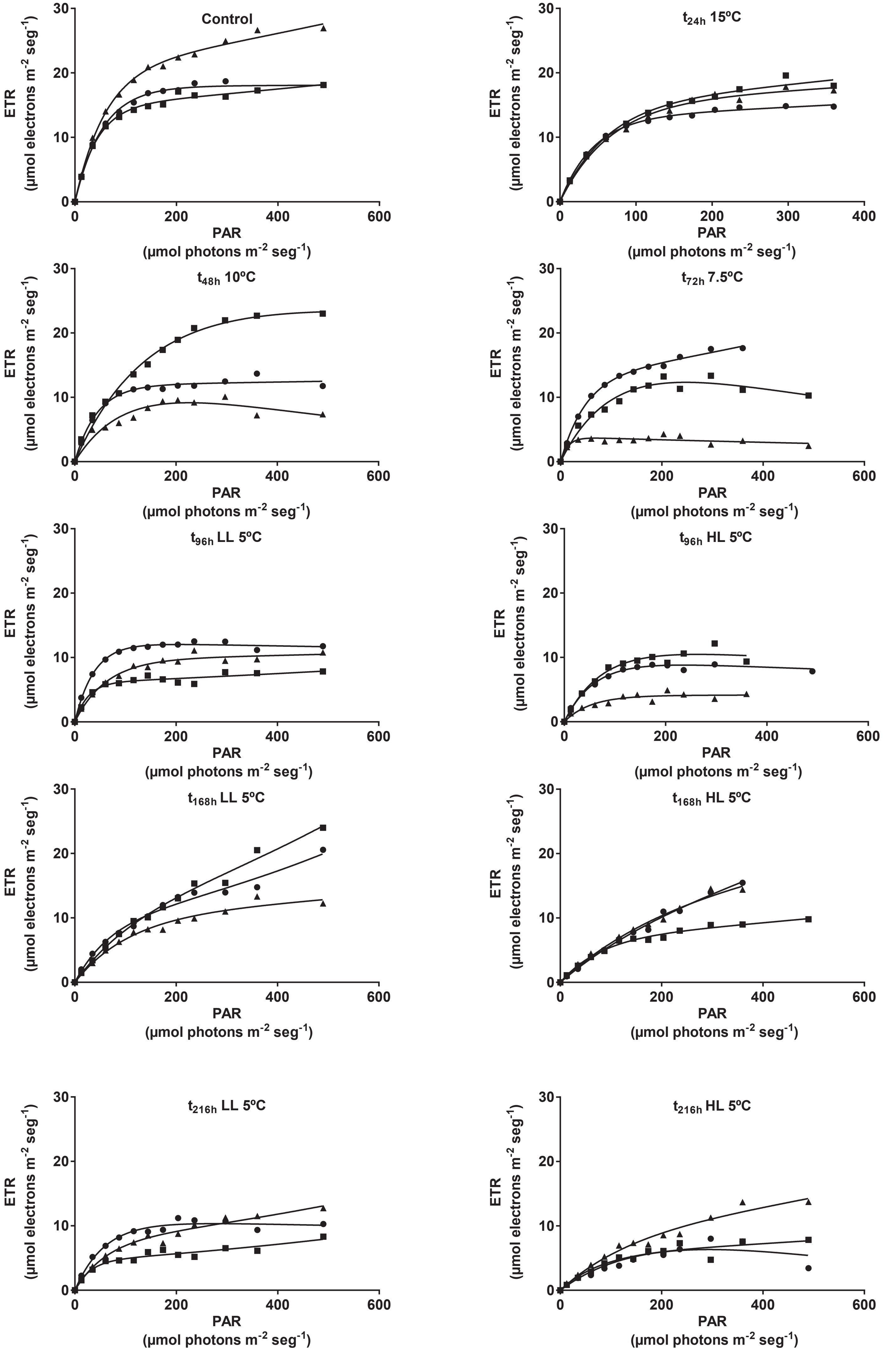

Apatococcus lobatus

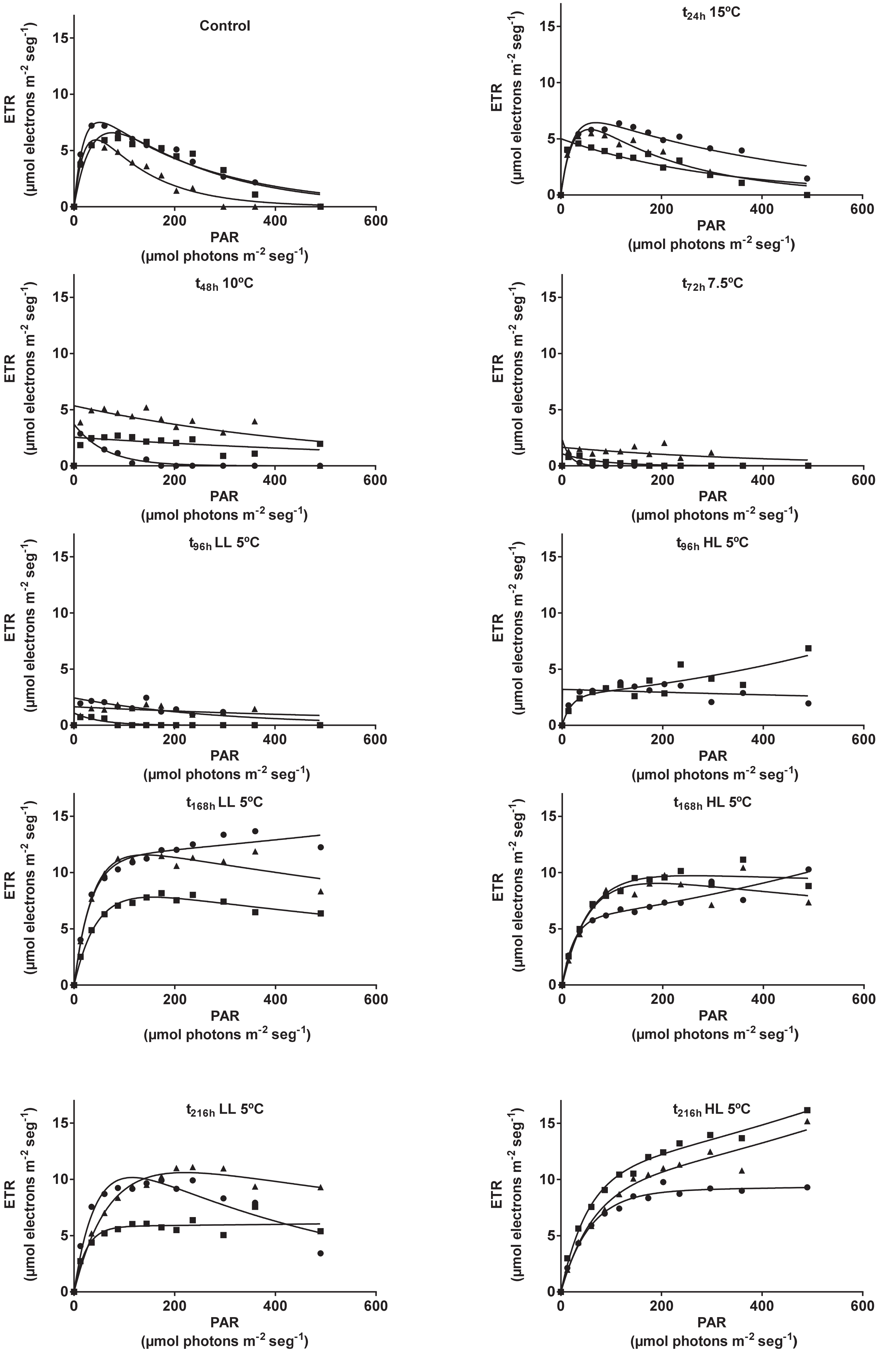

Ramalina pollinaria

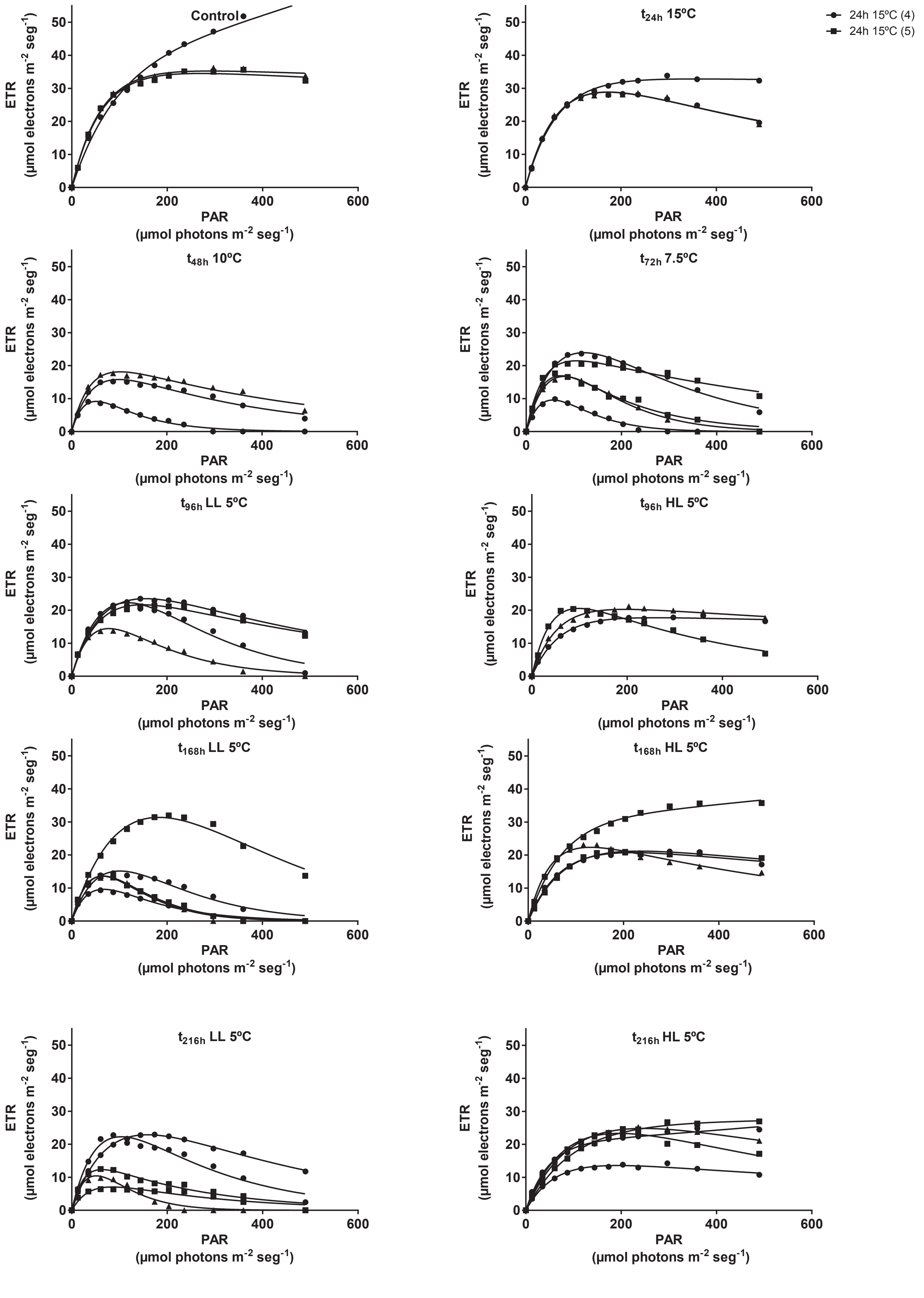

Trebouxia arboricola

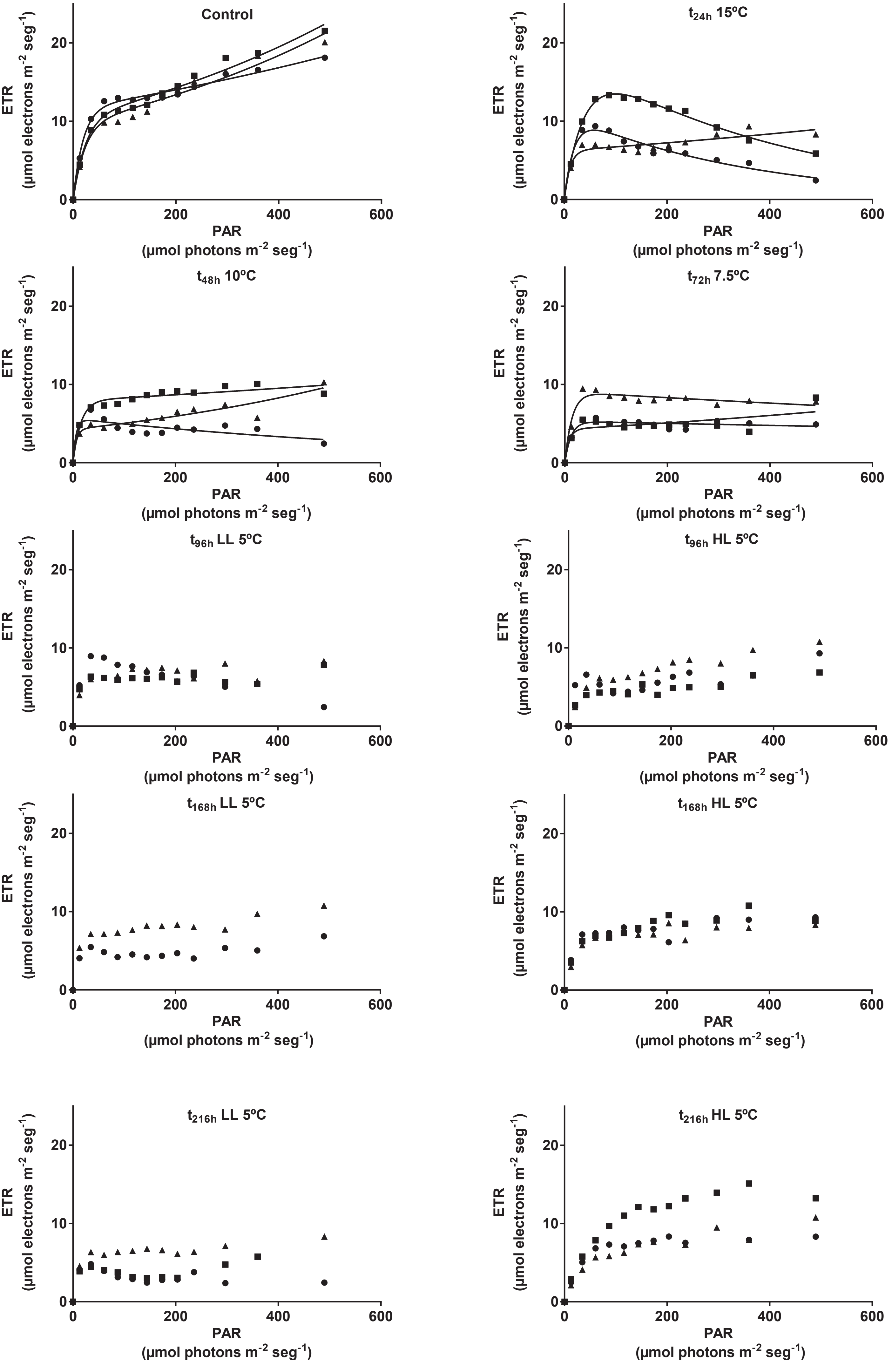

Elliptochloris bilobata

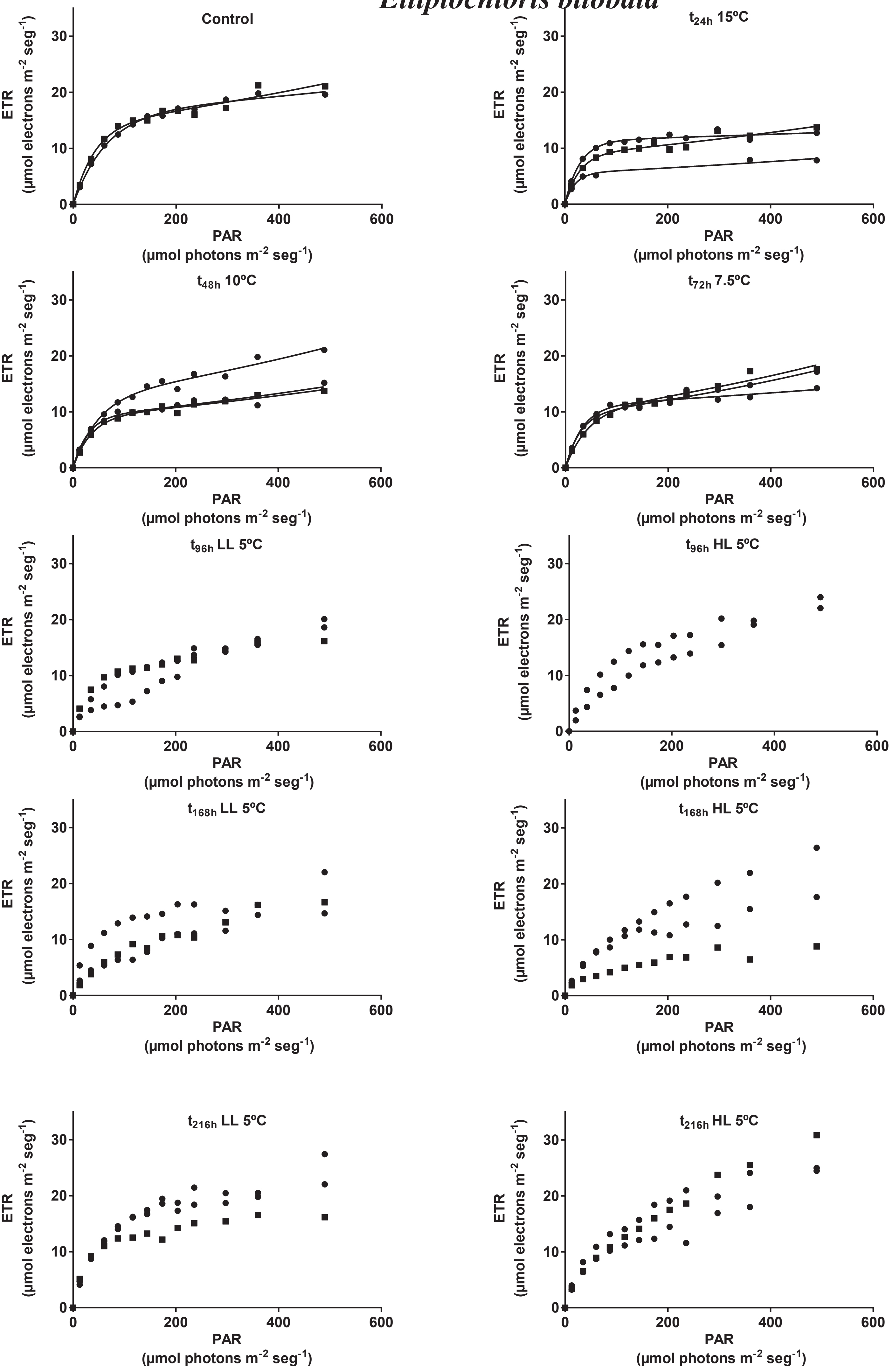

Supplement: Supplementary file 2 [file Presentation2.PDF]
