# Supplementary material for: Unraveling the Photoprotective Response of Lichenized and Free-Living Green Algae (Trebouxiophyceae, Chlorophyta) to Photochilling Stress
Source: Front Plant Sci. 2017 Jul 4;8:1144. doi: 10.3389/fpls.2017.01144 (PMC5495867; doi:10.3389/fpls.2017.01144)
Supplement: Supplementary file 3 [file Presentation3.PDF]

APPENDIX S3

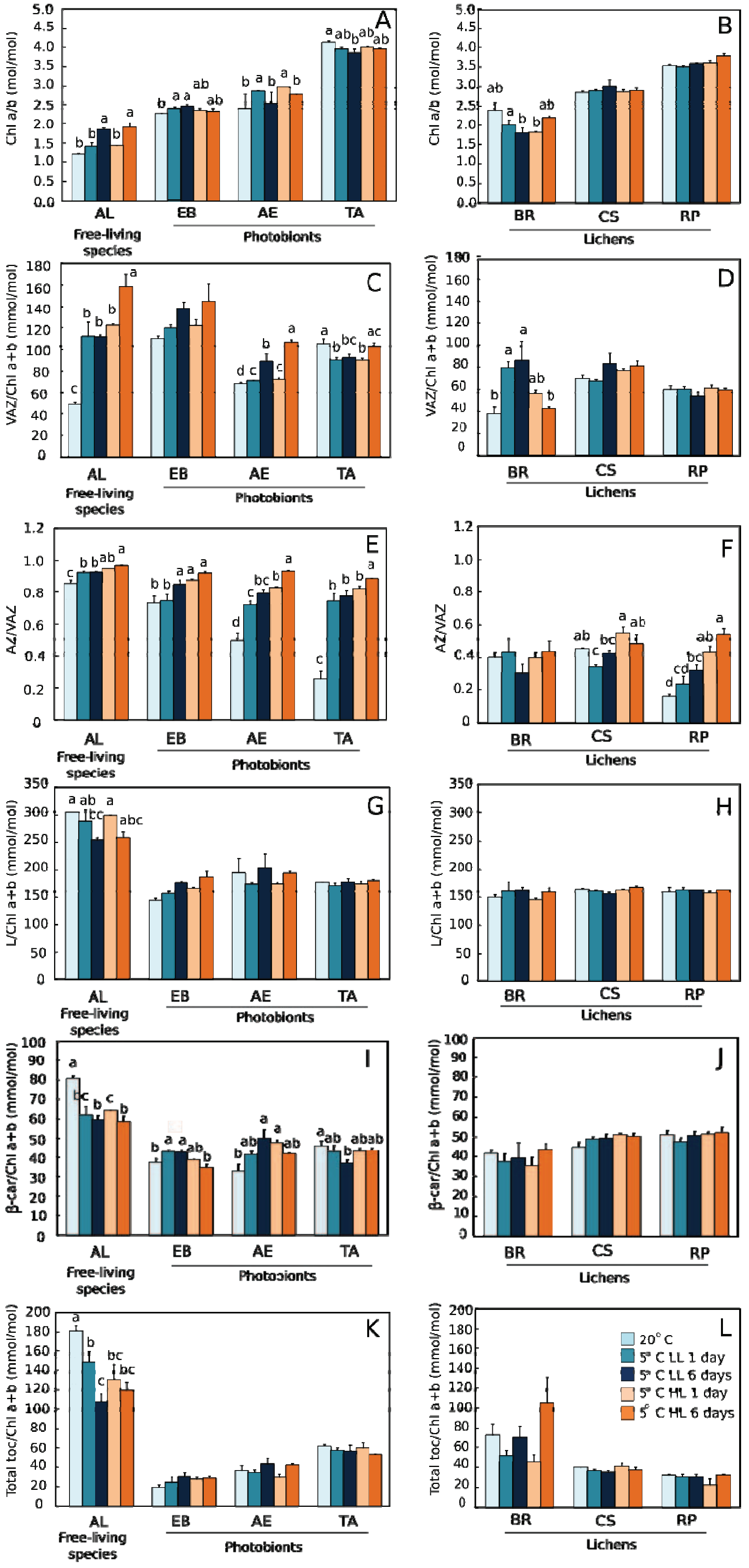

Effects of experimental treatments on pigment composition in free algae (left panels) and lichens (right panels). AL: Apatococcus lobatus; EB: Elliptochloris bilobata; AE: Asterochloris erici; TA: Trebouxia arboricola; BR: Baeomyces rufus; CS: Cladonia squamosa; RP: Ramalina pollinaria. Panels A and B show Chl a/b ratios in algae and lichens respectively; panels C and D show VAZ/Chl; pannels E and F show AZ/VAZ; pannels G and H show Lutein/Chl; pannels I and J show β-car/Chl and pannels K and L show total tocopherol/Chl. The first bar (pale blue) indicates control values under 20ºC. Blue bars refer to LT (low temperature) treatment and orange bars refer to LT-HL (low temperature-high light) treatment. Data are mean ± SE (n=3). Letters indicate significant differences for each species at P<0.05. The absence of letters means no significant differences.

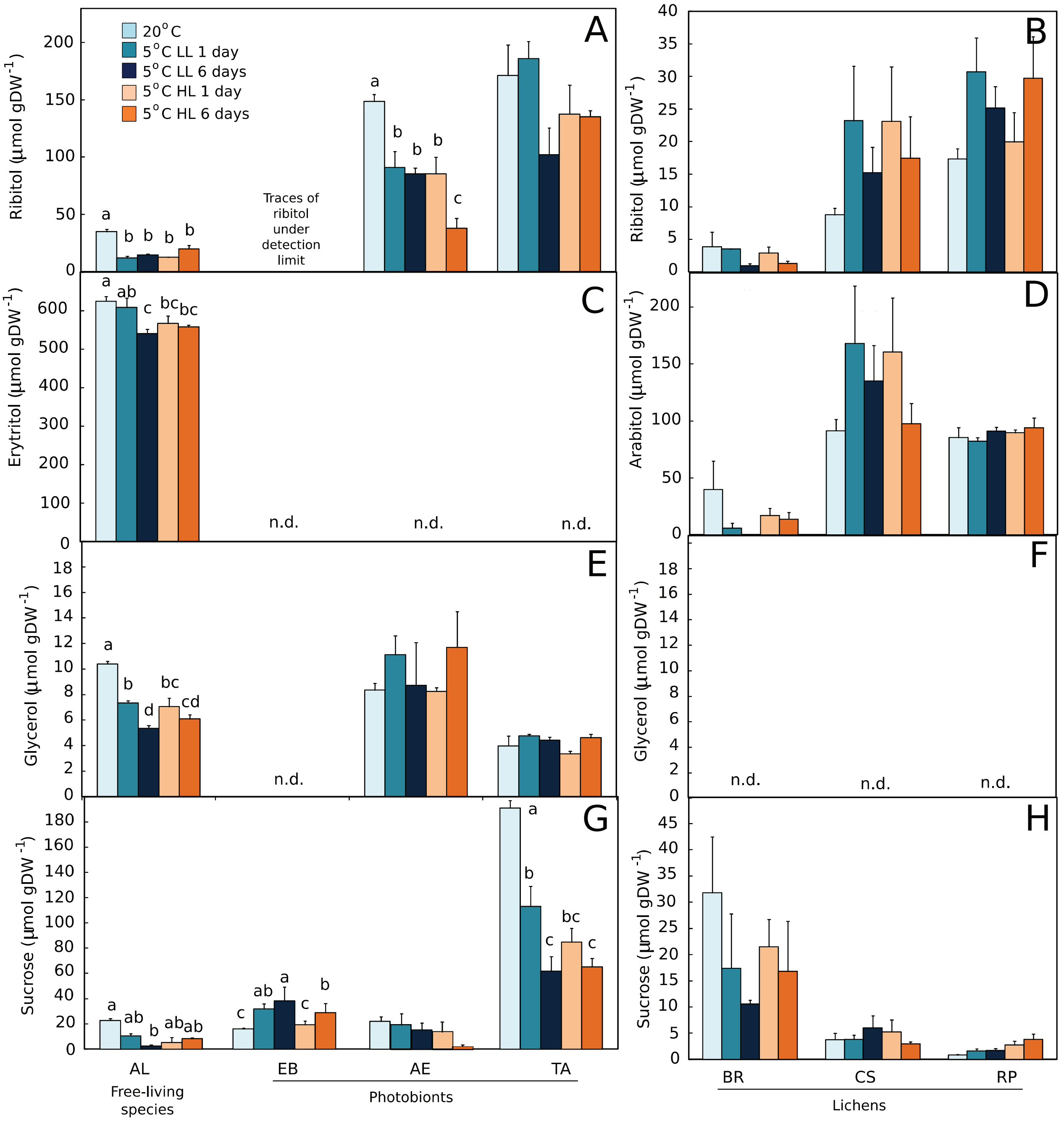

Effects of experimental treatments on low molecular weight carbohydrates (LMWC) in free algae (left panels) and lichens (right panels). *AL: Apatococcus lobatus; EB: Elliptochloris bilobata; AE: Asterochloris erici; TA: Trebouxia arboricola; BR: Baeomyces rufus; CS: Cladonia squamosa; RP: Ramalina pollinaria.* Panels A and B show ribitol content in algae and lichens respectively; panels C and D show erytritol content for algae and arabitol content for lichens respectively; panels E and F show glycerol content in algae and lichens respectively, and panels G and H show sucrose content in algae and lichens respectively. The first bar (pale blue) indicates control values under 20ºC. Blue bars refer to LT (low temperature) treatment and orange bars refer to LT-HL (low temperature-high light) treatment. All LMWC concentrations are given as µmol g-1 dry weight (DW). Data are mean ± SE (n=3).
