# Supplementary material for: Unraveling the Photoprotective Response of Lichenized and Free-Living Green Algae (Trebouxiophyceae, Chlorophyta) to Photochilling Stress
Source: Front Plant Sci. 2017 Jul 4;8:1144. doi: 10.3389/fpls.2017.01144 (PMC5495867; doi:10.3389/fpls.2017.01144)
Supplement: Supplementary file 4 [file Presentation4.PDF]

APPENDIX S4

Correlation coefficients (r) between de-epoxidation
state of violaxanthin cycle (AZ/VAZ) and non photochemical quenching (NPQ). Asterisks denote significant correlations (*: < 0.05; **: p < 0.01) and hyphen `-´ means no correlation.

**Algae**	TA	0.707**
	AE	-
	EB	-
	AL	-
**Lichens**	RP	0.557*
	CS	-
	BR	-
